# Analytical validation of a standardized scoring protocol for Ki67: phase 3 of an international multicenter collaboration

**DOI:** 10.1038/npjbcancer.2016.14

**Published:** 2016-05-18

**Authors:** Samuel C Y Leung, Torsten O Nielsen, Lila Zabaglo, Indu Arun, Sunil S Badve, Anita L Bane, John M S Bartlett, Signe Borgquist, Martin C Chang, Andrew Dodson, Rebecca A Enos, Susan Fineberg, Cornelia M Focke, Dongxia Gao, Allen M Gown, Dorthe Grabau, Carolina Gutierrez, Judith C Hugh, Zuzana Kos, Anne-Vibeke Lænkholm, Ming-Gang Lin, Mauro G Mastropasqua, Takuya Moriya, Sharon Nofech-Mozes, C Kent Osborne, Frédérique M Penault-Llorca, Tammy Piper, Takashi Sakatani, Roberto Salgado, Jane Starczynski, Giuseppe Viale, Daniel F Hayes, Lisa M McShane, Mitch Dowsett

**Affiliations:** 1 Department of Pathology and Laboratory Medicine, University of British Columbia, Vancouver, British Columbia, Canada; 2 Academic Department of Biochemistry, Royal Marsden Hospital and Institute of Cancer Research, London, United Kingdom; 3 Department of Pathology, Tata Medical Center, Kolkata, West Bengal, India; 4 Department of Pathology and Laboratory Medicine, Indiana University Simon Cancer Center, Indianapolis, Indiana, USA; 5 Department of Pathology and Molecular Medicine, Juravinski Hospital and Cancer Centre, McMaster University, Hamilton, Ontario, Canada; 6 Transformative Pathology, Ontario Institute for Cancer Research, Toronto, Ontario, Canada; 7 Department of Clinical Sciences, Division of Oncology and Pathology, Lund University, Lund, Sweden; 8 Department of Pathology, Mount Sinai Hospital, Toronto, Ontario, Canada; 9 The EMMES Corporation, Rockville, Maryland, USA; 10 Department of Pathology, Montefiore Medical Center and the Albert Einstein College of Medicine, Bronx, New York, USA; 11 Department of Pathology, Dietrich-Bonhoeffer Medical Center, Neubrandenburg, Mecklenburg-Vorpommern, Germany; 12 PhenoPath Laboratories, Seattle, Washington, USA; 13 Lester and Sue Smith Breast Center and Dan L. Duncan Cancer Center, Baylor College of Medicine, Houston, Texas, USA; 14 Department of Laboratory Medicine and Pathology, University of Alberta, Edmonton, Alberta, Canada; 15 Department of Pathology and Laboratory Medicine, The Ottawa Hospital, Ottawa, Ontario, Canada; 16 Department of Pathology, Slagelse Hospital, Slagelse, Region Sjælland, Denmark; 17 Fred Hutchinson Cancer Research Center, Seattle, Washington, USA; 18 Division of Pathology and Laboratory Medicine, European Institute of Oncology, Milan, Italy; 19 Department of Pathology, Kawasaki Medical School, Kurashiki, Okayama Prefecture, Japan; 20 Department of Laboratory Medicine, Sunnybrook Health Sciences Centre, Toronto, Ontario, Canada; 21 Department of Pathology, Centre Jean Perrin and Université d'Auvergne, Clermont-Ferrand, France; 22 Biomarkers & Companion Diagnostics Group, Edinburgh Cancer Research Centre, Western General Hospital, Edinburgh, United Kingdom; 23 Department of Pathology, Nippon Medical School, Bunkyo-ku, Tokyo, Japan; 24 Breast Cancer Translational Research Laboratory, Institut Jules Bordet, Brussels, Belgium; 25 Department of Cellular Pathology, Birmingham Heart of England, National Health Service, Birmingham, United Kingdom; 26 Division of Pathology and Laboratory Medicine, European Institute of Oncology and University of Milan, Milan, Italy; 27 Breast Oncology Program, Department of Internal Medicine, University of Michigan Comprehensive Cancer Center, Ann Arbor, Michigan, USA; 28 Biometric Research Branch, Division of Cancer Treatment and Diagnosis, National Cancer Institute, Bethesda, Maryland, USA

## Abstract

Pathological analysis of the nuclear proliferation biomarker Ki67 has multiple potential roles in breast and other cancers. However, clinical utility of the immunohistochemical (IHC) assay for Ki67 immunohistochemistry has been hampered by unacceptable between-laboratory analytical variability. The International Ki67 Working Group has conducted a series of studies aiming to decrease this variability and improve the evaluation of Ki67. This study tries to assess whether acceptable performance can be achieved on prestained core-cut biopsies using a standardized scoring method. Sections from 30 primary ER+ breast cancer core biopsies were centrally stained for Ki67 and circulated among 22 laboratories in 11 countries. Each laboratory scored Ki67 using three methods: (1) global (4 fields of 100 cells each); (2) weighted global (same as global but weighted by estimated percentages of total area); and (3) hot-spot (single field of 500 cells). The intraclass correlation coefficient (ICC), a measure of interlaboratory agreement, for the unweighted global method (0.87; 95% credible interval (CI): 0.81–0.93) met the prespecified success criterion for scoring reproducibility, whereas that for the weighted global (0.87; 95% CI: 0.7999–0.93) and hot-spot methods (0.84; 95% CI: 0.77–0.92) marginally failed to do so. The unweighted global assessment of Ki67 IHC analysis on core biopsies met the prespecified criterion of success for scoring reproducibility. A few cases still showed large scoring discrepancies. Establishment of external quality assessment schemes is likely to improve the agreement between laboratories further. Additional evaluations are needed to assess staining variability and clinical validity in appropriate cohorts of samples.

## Introduction

Assessment of the nuclear proliferation biomarker Ki67 has multiple potential roles in breast and other cancers,^1,2^ either in standard clinical practice as a prognostic^3–11^ and predictive^5,7,10,12^ marker or in clinical trials as an eligibility criterion or as a primary end point in early-phase neoadjuvant studies.^10^ Perhaps the most critical use for standard clinical care would be to determine prognosis in the context of other factors, such as nodal status, tumor size, and estrogen receptor, progesterone receptor, and HER2 status. Although gene expression multiparameter molecular assays have gained widespread use in the United States and other countries, these assays may not be an option in many clinical settings owing to availability or economic considerations. Therefore, the Ki67 immunohistochemistry assay might offer a cost-effective alternative.^13–15^ The 2015 St Gallen consensus panel stated that the majority of new breast cancer cases and breast cancer deaths now occur in less developed regions of the world,^13^ accentuating the need for low cost, widely accessible biomarkers. However, despite extensive effort spent on evaluating Ki67 as a prognostic and/or predictive marker in the past three decades, this biomarker is still not completely integrated into clinical decision making,^16^ due mainly to the lack of standardization in staining techniques and scoring methods.^3,10,16^

The International Ki67 Working Group has undertaken a systematic multiphase program to determine whether Ki67 scoring can be analytically validated and standardized across laboratories.^10,17,18^ In phase 1, variability in visual interpretation, as assessed by the intraclass correlation coefficient (ICC) estimate of interobserver reproducibility, was the most important source of variability (ICC=0.71, 95% credible interval (CI): 0.47–0.78).^17^ In phase 2, substantial levels of agreement were achieved when the various laboratories followed clearly defined, standardized training exercise and scoring methods.^18^ Indeed, the interobserver variability observed in phase 2 (ICC=0.94, 95% CI: 0.90–0.97) is similar to the intraobserver reproducibility (ICC=0.94; 95% CI: 0.93–0.97) observed in phase 1. However, this level of agreement was achieved when scoring the same tumors on tissue microarrays, whereas in clinical practice biomarker decisions are made on core-cut biopsy or on surgical excision whole-section specimens. Such specimens require pathologists to select specific regions for assessment within a larger area, and so increased variability in scoring would be expected.

Given the encouraging result achieved on breast cancer tissue microarrays, we proceeded to phase 3 to assess whether acceptable performance can be achieved on core-cut biopsies using a similar, standardized method including two distinct approaches in selecting which area to score.

## Results

### Interlaboratory ICC concordance of Ki67 according to method of scoring

**Figure 1:**
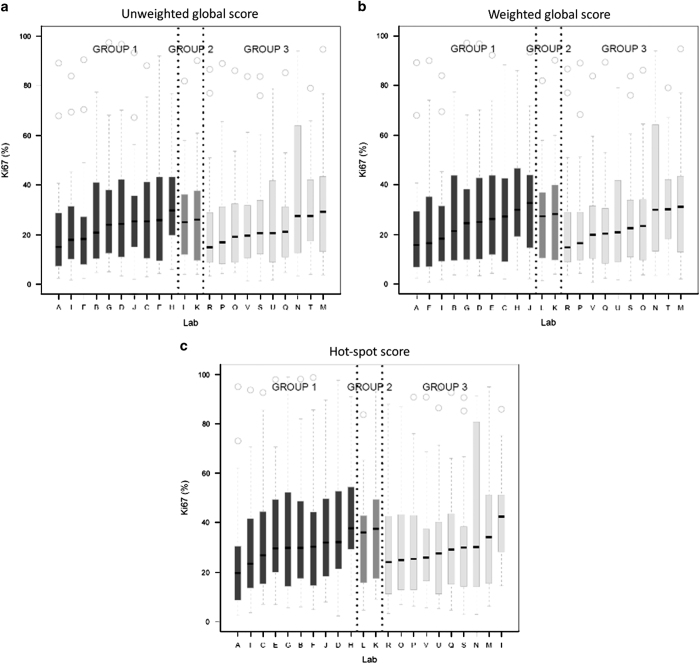
Ki67 scores (**a**, unweighted global; **b**, weighted global; **c**, hot-spot) of all 22 laboratories (by group): black for Group 1, medium gray for Group 2, and light gray for Group 3. Laboratories are ordered (within each group) by the median scores. The bottom/top of the box in each box plot represent the first (Q1)/third (Q3) quartiles, the bold line inside the box represents the median and the two bars outside the box represent the lowest/highest datum still within 1.5×the interquartile range (Q3–Q1). Outliers are represented with empty circles.

The different-section ICC estimate for the unweighted global score was 0.87 (95%CI: 0.81–0.93), and therefore met the prespecified success criterion (lower bound of credible interval exceeding 0.8; Table 1). The different-section ICCs for the weighted global score and hot-spot score were 0.87 (95%CI: 0.7999–0.93) and 0.84 (95%CI: 0.77–0.92), respectively, and therefore both methods had ICC credible intervals that extended below the success criterion. The corresponding same-section ICC estimates for the unweighted global, weighted global and hot-spot scores were 0.88 (95% CI: 0.81–0.93), 0.87 (95% CI: 0.80–0.93) and 0.84 (95% CI: 0.77–0.92), respectively. Figure 1 displays the side-by-side boxplots of Ki67 scores across laboratories by group. Summary statistics for the Ki67 scores across the 22 laboratories are given in Supplementary Tables 2.

**Figure 2:**
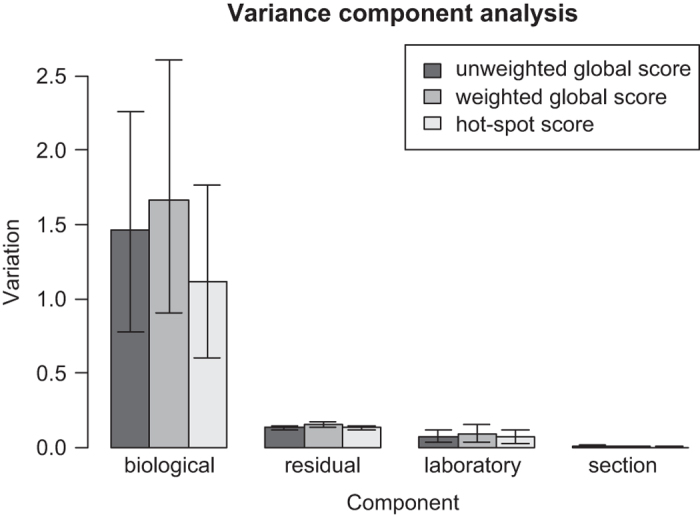
Variance component analysis. Variation due to different components are presented in a bar plot to show the relative magnitude differences between them. Numeric values of the variance components estimates and the corresponding credible intervals are shown in Supplementary Table 5.

Variance component analyses show that, regardless of scoring method, biological variation among different patients was the largest component of the total variation, indicating that the Ki67 score is reflecting inherent properties of the tumor and that the effect on the score introduced by the immunohistochemistry assay technical variation (sectioning, staining, and scoring) is relatively small (Figure 2, Supplementary Table 5).

### Interlaboratory variation of Ki67 scoring

**Figure 3:**
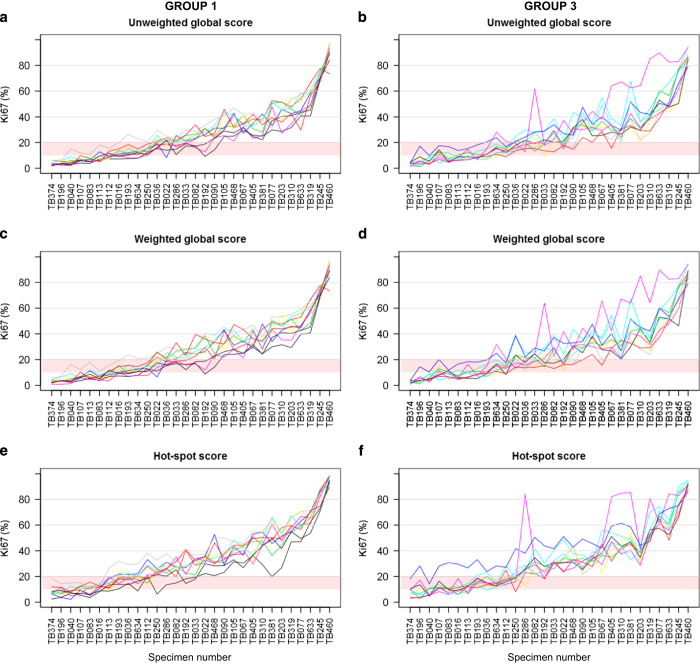
Variability in Ki67 scores (**a**, **c** and **e** correspond to Group 1; **b**, **d** and **f** correspond to Group 3). Each line represents Ki67 scores from one laboratory. Shaded region indicates Ki67 scores between 10 and 20%. Scores from Group 2 are not shown since there are only two laboratories in this group.

**Figure 4:**
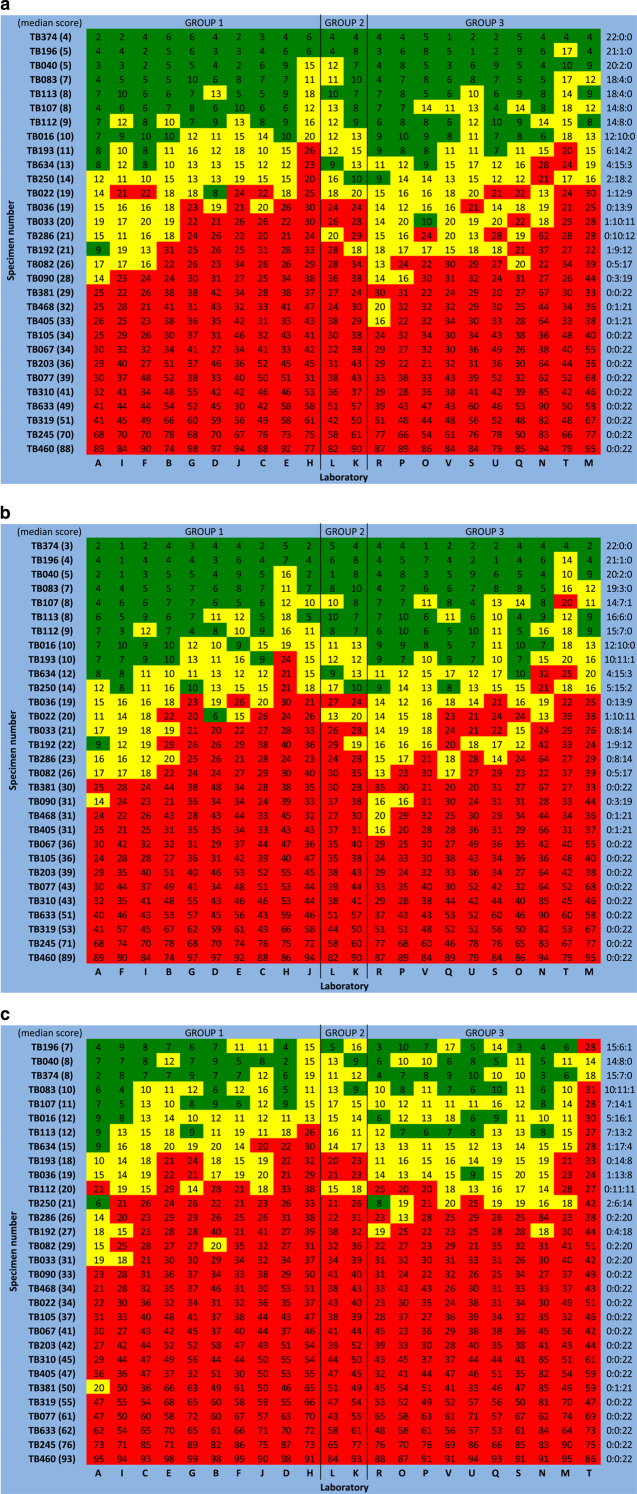
Heat map of Ki67 scores (**a**: unweighted global; **b**: weighted global; **c**: hot-spot). Rows represent cases and columns represent laboratories. Green color indicate that the score is <10%, yellow 10–20%, and red >20%. Cases are ordered by the median scores (across laboratories), which are shown in parentheses beside the specimen number. Laboratories are ordered (within each group) by the median scores (across cases). The three colon-separated numbers to the right of the table represent the number of laboratories giving scores falling into different ranges: <10% (left-most), 10–20% (middle) and >20% (right-most). For example, ‘15:6:1’ indicates that 15 laboratories gave a score of <10%, six laboratories between 10 and 20% and one laboratory >20%.

Figure 3 displays the variation in scores across laboratories for each case, in spaghetti plot format. Each line represents scores from one laboratory. Figure 4 presents the scores in a heat map format with the columns (laboratories) sorted (within each group) by the median scores across cases and the rows (cases) sorted by the median scores across laboratories.

Overall it can be seen that most laboratories show good parallelism in the increasing Ki67 scores across the plots. In other words, laboratories measuring higher or lower than others tended to do so relatively consistently. In group 3 one lab (N) can be seen to score considerably higher than the others in both the unweighted and weighted scores particularly in the samples with the higher scores. This laboratory also showed a number of higher scores on the hot-spot method. Another laboratory in group 3 (T) also showed substantially and consistently higher hot-spot scores than the others, whereas in group 1 one laboratory (A) can be seen to score consistently lower than the others.

### Categorical concordance of Ki67 scoring

With regard to agreement on a categorical level (rather than on a continuous, 0–100% scale), considering the categories <10%, 10–20%, and >20%, the relationship between percent agreement and continuous score is shown in Supplementary Figure 3. It shows excellent to perfect agreement on cases with scores that are either much lower or higher than the intermediate range of 10–20%.

**Figure 5:**
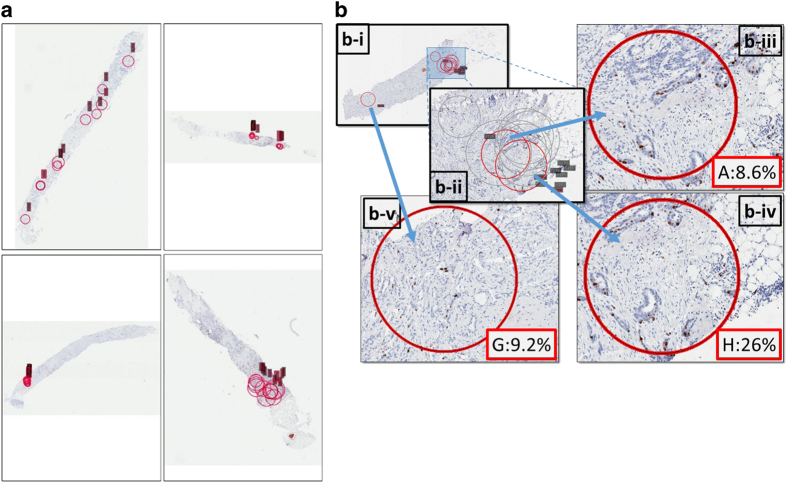
Hot-spot field selection by different laboratories on the same core-cut biopsy slide. (**a**) Selections (indicated by red circles) on some example core biopsies. (**b**) Example of a single-core biopsy (median score: 12%) with zoomed-in fields. Each laboratory was asked to circle the area considered by that laboratory to be the hot-spot (**b**-**i**). Most pathologists honed in on the same area of the core, although individual-selected circular scoring fields do not always overlap. (**b-iii**, **b-iv**) Segments of the same area chosen by two different laboratories to read Ki67. (**b-v**) The ‘outlier’ field selected by only one laboratory as the hot-spot.

Visually, there was moderately strong agreement across laboratories in the pathologist-selected location of the hot-spots in each of the core-cut biopsies (Figure 5 shows some examples; virtual slide images of all core-cut biopsy slides used in this study and the corresponding selected fields and scores can be viewed at http://www.gpec.ubc.ca/papers/ki67p3).

After selection of the fields to score, the median times required for nuclei counting were 3 and 4 min for the global and hot-spot methods, respectively.

## Discussion

The overarching goal of the International Ki67 Working Group multiphase program is to build enough evidence to either support or refute the notion that Ki67 assessed by immunohistochemistry is sufficiently analytically and clinically validated to be implemented in routine clinical practice for management of breast cancer.^10^ Our previous studies demonstrated substantial interlaboratory variability in Ki67 scoring among some of the world’s leaders in the field, even when reading centrally stained slides (phase 1).^17^ However, this variability was reduced by introducing a standardized, practical visual scoring method (phase 2)^18^—a method that does not require any special equipment beyond a desktop computer and light microscope.

In this third study, we progressed to a more ‘real world’ circumstance of reading Ki67 staining of core biopsies, while still controlling for variability due to preanalytical and analytical aspects of the assay.^10^ We have demonstrated that it is possible, given a set of clearly defined training exercise and scoring instructions, for pathologists to achieve high interobserver agreement in scoring Ki67 on core-cut biopsies using a conventional light microscope and manual field selection, with no additional aid such as counting grid or software. The average time taken to score, once fields for scoring had been selected, was between 3 and 4 min regardless of the method and was judged to be acceptable in general practice by the participants in the current study.

We found that the global unweighted method achieved the observed highest ICC and CI (0.87, 95% CI: 0.81–0.93) compared with weighted global (0.87, 95% CI: 0.7999–0.93) and hot-spot methods (0.84, 95% CI: 0.77–0.92). Thus the global method was the only one to meet the prespecified criterion of success but the other methods missed this criterion by a small amount and cannot be ruled out as viable alternatives. The results do not provide sufficient evidence that the global method is significantly more reproducible, as measured by ICC, than the others. There appeared to be moderately strong agreement in the location of the selected hot-spots across laboratories (Figure 5a). However, as shown in Figure 5b, even a very slight difference in hot-spot location could result in a large difference in the Ki67 scores (8.6% vs. 26%). Our findings are in agreement with other reports, in which a marginally higher concordance among global compared with hot-spot scores was observed (ICC=0.904 vs. ICC=0.894, respectively).^19^ We propose that differences in individual fields average out in the global method, and thus the overall score is more robust to variability introduced by the exact localization of the fields selected for scoring.

Despite the conclusion that the scoring aspect of analytical validity has been achieved based on overall assessment by ICC, there are still a few cases with large discrepancies (Figures 4a–c). To understand potential sources of these variabilities, a subsequent exploratory examination of the field selections and scores on individual fields were performed (Supplementary Document: ‘Exploratory examination of scoring fields’). Five sources of variability were identified: (1) scoring of ductal carcinoma *in situ* tissue; (2) scoring of stromal cells; (3) positive nuclei being localized within a different part of the selected field; (4) need for recalibration; (5) different hot-spots within a single slide exhibiting different Ki67 scores. Some of these factors may be correctable/preventable (1, 2, 4) while others may be difficult to avoid (3, 5).

Among the rest of the cases, much of the scoring variability that remains between laboratories relates to relatively consistent low or high bias for scorers. Establishment of external quality assessment schemes and regular participation in such programs may improve the agreement between laboratories further, especially in the area of standardizing staining protocol (which has not yet been addressed in any of our studies).

Similar to what was observed in phase 2,^18^ clinically important discrepancies persisted among laboratories for some cases in the intermediate Ki67 range between 10 and 20% (Supplementary Figure 4). These discrepancies are of concern, since this is the range in which cutoffs to distinguish high from low Ki67 levels are usually selected and used to make clinical decisions.^6,13,20^ There were 19 cases for which at least one of the 22 laboratories reported an unweighted global score in the range of 10%⩽Ki67⩽20%. Strikingly, there were no cases where all laboratories provided scores that were confined to this range. If the intermediate Ki67 range extends to 10%⩽Ki67⩽30%, then there were 26 cases for which at least one of the 22 laboratories reported an unweighted global score in this range. There was only one case for which all laboratories provided scores in this extended cutoff range.

On the other hand, as evident from the heat maps (Figures 4a–c), exceptionally high to unanimous agreement was observed for cases with median Ki67 scores that were either much higher or lower than the intermediate range (10%⩽Ki67⩽20%): 100% agreement was observed with the global method (unweighted and weighted) on 11/30 (37%) cases and, with hot-spot method, 13/30 (43%) cases. Supplementary Figure 3 shows the relationship between scores and the rate of agreement on categories. It demonstrates that there is often some disagreement, but for scores that are far away from the intermediate range, for example, above 35%, everyone agreed. However, at lower levels there was increasing disagreement.

Ki67 IHC might be used for one of many possible applications, including for determination of breast cancer intrinsic subtype,^21^ use in IHC-based multiparameter assays to approximate results from gene expression assays such as the 21-gene recurrence score,^14,15^ and use in IHC-based prognostic models.^22,23^ Regardless, Ki67 is usually interpreted in the context of other clinicopathological parameters, such as tumor size, lymph node status and grade, or biomarkers, such as ER, PR and HER2 status. In this regard, Denkert *et al.* noted that treatment decisions for individual patients should not be made based on small differences of Ki67 around a given cutpoint.^16^ Further studies in the impact of Ki67 scoring variability on multiparameter clinical application would be beneficial. Regardless, the increasing scoring concordance we have observed through our three phases of consensus training suggests some progress toward Ki67 immunohistochemistry applicability in the standard of care setting, assuming proper training and adherence to proficiency testing.

While our study shows that Ki67 visual scoring systems can be standardized to reach high levels of interobserver agreement (as measured by ICC) in centrally stained core-cut biopsy samples, it has several limitations. In clinical practice, additional preanalytical and analytical aspects, such as staining protocol differences,^10^ will add substantial variability, as will moving from core-cut biopsies onto whole sections. In addition, the clinical validity (and therefore clinical utility) of this specific scoring system has yet to be confirmed. The data from the current phase 3 study are sufficiently positive to support proceeding to evaluation of these other aspects by consortium members in a series of planned studies.

In conclusion, we believe we are one step closer to standardizing the Ki67 immunohistochemistry assay for use in breast cancer. However, at this stage, we cannot yet recommend this assay platform to be used to drive patient-care decisions in clinical practice.

## Materials and methods

This study was approved by the British Columbia Cancer Agency Clinical Research Ethics Board (protocol H10-03420). All samples used in this study were donated by patients who signed a generic consent. All core-cut biopsy material used in this study was excess to diagnostic requirements and ethically available for quality control studies.

### Case selection

One hundred and ten cases of estrogen receptor (ER) positive breast cancer were selected from the Academic Department of Biochemistry (ADB) tumor bank at the Royal Marsden Hospital, UK. Sixty-nine of these were further selected for initial sectioning, based on visual estimation of the available material, Haematoxylin and Eosin (H&E) and Ki67 staining using Academic Biochemistry protocols.^24^ Quality of each section (for example, crush artifacts and cellularity) was assessed and the percentage of Ki67 positivity was estimated. A set of 40 core-cut biopsy blocks was sectioned and stained in the Royal Marsden Hospital Histopathology Department using monoclonal antibody MIB1 at dilution 1:50 (DAKO UK, Cambridgeshire, UK) using an automated staining system (Ventana Medical Systems, Tucson, AZ, USA) according to the criteria established by consensus of the International Ki67 Working Group.^10^ The final set of 30 core-cut biopsy sections was selected on the basis of sufficient cell numbers and quality of staining (Supplementary Figure 1). The distribution of clinicopathological parameters among these 30 cases is shown in Supplementary Table 1.

### Sample preparation and distribution

Twenty-four volunteer laboratories, most of whom participated in phase 1 or 2 of the International Ki67 Working Group initiatives, representing 23 institutions from 11 countries, were invited to participate in phase 3.

Five adjacent sections from each of the 30 core-cut biopsy source blocks were centrally stained. The first section was stained with H&E, the second with a myoepithelial marker (p63) and the third to fifth sections with Ki67. Because the time required to have all laboratories review the same slide would have been prohibitive, the latter three Ki67-stained sections were prepared and are designated Groups 1, 2, and 3. Each group of slides included 30 sections, one from each of the 30 patients. The participating laboratories were initially divided into three groups (eight laboratories in each group) and members within the same group were given the same group of slides to score. Because the slides were damaged en route to the third volunteer laboratory in Group 2, members within this group who had not yet scored were subsequently reassigned to Group 1 or 3. Two volunteer laboratories did not complete the study in time for the analysis. Twenty-two laboratories successfully completed the study: 10 laboratories in Group 1, two in Group 2 and 10 in Group 3.

### Scoring protocol

All laboratories were required to complete the phase 2 web-based calibration exercise^18^ prior to the phase 3 scoring. This calibrator is publicly accessible at http://www.gpec.ubc.ca/calibrator. The detailed scoring protocol is found in Supplementary Document: ‘Instructions for Ki67 Reproducibility Study Phase 3: Core Biopsies’. A modified version of the scoring software (modified for offline use) used in this study can be downloaded at: http://www.gpec.ubc.ca/papers/ki67p3.

### Scoring methods

Three scoring methods were assessed in this study: (1) an unweighted global assessment of Ki67 staining; (2) a global assessment that is weighted according to the estimated percentage of the total cancer area covered by each of high, medium, low, or negligible Ki67 staining levels and (3) assessment of Ki67 only in ‘hot-spots.’

Global methods attempt to derive an average score across all the tissue available for assessment. In the weighted and unweighted global methods, Ki67 index counting was performed in the same manner, but the final Ki67 score was derived differently. Adapted from a scoring protocol that has been used routinely in the Dowsett ADB laboratory,^24^ these two global methods require the pathologist to first assess staining heterogeneity by estimating the percentages of the invasive tumor component of the slide exhibiting relatively high, medium, low or negligible Ki67 scores. On the basis of these estimates, a standard algorithm (Supplementary Figure 2) determined the required number of fields to score for each Ki67 score level (total up to four fields). The pathologist was then asked to count up to 100 invasive tumor nuclei within each field, using a ‘typewriter’ pattern, similar to how a tissue microarray core was scored in the phase 2 study.^18^

Variations on hot-spot assessments are often used by pathologists for mitotic counting, where the pathologist identifies what appears to be the most active area of cell division. The hot-spot method required the pathologist to select one high-power field with high staining rate and count up to 500 invasive tumor nuclei in a ‘typewriter’ pattern.

### Statistical analyses

#### Prespecified criterion for success

Prior to data collection, it was hypothesized that at least one of the scoring methods would have an associated ICC of at least 0.80. For planning purposes, power calculations performed under a variety of scenarios considered to represent good reproducibility and similar to the results observed in the phase 2 study showed that with 21 laboratories there would be 80% power to exclude ICCs lower than the prespecified ICC of 0.8 from a 95% credible interval for a given scoring method. This success criterion of ‘0.8’ was chosen using criteria similar to those for Kappa value interpretation: 0.81–1 indicating ‘almost perfect’ agreement.^25^

#### Ki67 scoring

The Ki67 score was defined as the percentage of invasive tumor cells positively stained in the examined field(s). Positive staining was defined as any brown stain in the nucleus above background, illustrated by sample images; negative staining was scored when the invasive cancer cell showed only a blue counterstained nucleus. The unweighted global and hot-spot scores were simply the total number of positively stained tumor nuclei counted divided by the total number of tumor nuclei counted (in the one hot-spot field, or across all fields for the global method). The weighted global score was derived with tumor nuclei counts in each assessed field weighted by the estimated percentage of the total cancer area covered by each of high, medium, low, or negligible Ki67 staining levels. For example, consider a slide estimated to have 10% of its area covered by relatively high Ki67 index regions, while 90% of the area is covered by relatively low-level Ki67 regions. By the algorithm (Supplementary Figure 2), the required number of fields to select and score is one high field and three low fields. Suppose the number of positive/total tumor nuclei counted in the high field is 85/100 and the three low fields are 30/100, 20/80, and 18/90. The weighted Ki67 score would be 0.1×(85/100)+0.9×((30+20+18)/(100+80+90))=31%, whereas the unweighted score would be (85+30+20+18)/(100+100+80+90)=41%. For the statistical analysis, the Ki67 score was transformed to a logarithmic scale by adding 0.1% and applying a log base 2 transformation to satisfy model assumptions of normality and constant variance.^10^

ICC estimates (ranging from 0 to 1, with 1 representing perfect reproducibility) were computed as previously reported in the phase 2 study.^18^ Briefly, variance component analyses were performed to quantify the contributions from the following sources of variability: scoring laboratory, patient tumor (biological variation—each core-cut biopsy block represents a unique patient) and section of the core-cut biopsy block. Similar to the phase 2 study, same-section and different-section ICC were computed. Same-section refers to scoring laboratories scoring the same set of core-cut biopsy slides, whereas different-section refers to scoring laboratories scoring different sections of the same core-cut biopsy blocks. CI for the variance components and the ICCs were obtained using the Markov Chain Monte Carlo routines for fitting generalized linear mixed models.

All data analyses were performed using R version 3.2.1.^26^ Sources of variation in log2-transformed Ki67 scores were analyzed using random effects models as implemented in the R packages lme4 and MCMCglmm. Data were visualized using heat maps, boxplots and spaghetti plots.

## Figures and Tables

**Table 1:** Summary of ICC values for different scoring methods

	*Different-section ICC*	*Same-section ICC*
Unweighted global	0.87 (95% CI: 0.81–0.93)	0.88 (95% CI: 0.81–0.93)
Weighted global	0.87 (95% CI: 0.7999–0.93)	0.87 (95% CI: 0.80–0.93)
Hot-spot	0.84 (95% CI: 0.77–0.92)	0.84 (95% CI: 0.77–0.92)

Abbreviations: CI, credible interval; ICC, intraclass correlation coefficient.
