# Supplementary material for: Analytical validation of a standardized scoring protocol for Ki67: phase 3 of an international multicenter collaboration
Source: NPJ Breast Cancer. 2016 May 18;2:16014–. doi: 10.1038/npjbcancer.2016.14 (PMC5515324; doi:10.1038/npjbcancer.2016.14)
Supplement: Supplementary Document 1 [file npjbcancer201614-s1.doc]

**Supplemental figure 1.** Case selection diagram.

**Supplemental figure 2.** Algorithm for allocating the required number of fields to score for global methods.

**Supplemental figure 3. Percent agreement on categories (<10%, 10–20%, >20%) over Ki67 scores. For each of the 30 cases, percentage of laboratories giving a score that falls into the same category as the median score is plotted against the median score. The dotted lines indicate 10 and 20%. Locally-weighted polynomial regression (performed using the R function “lowess” from the stats package^1,2^; function was applied to log2-transformed data with default parameters) has been applied to highlight the general trend. Empty circles represent actual data points.**

| **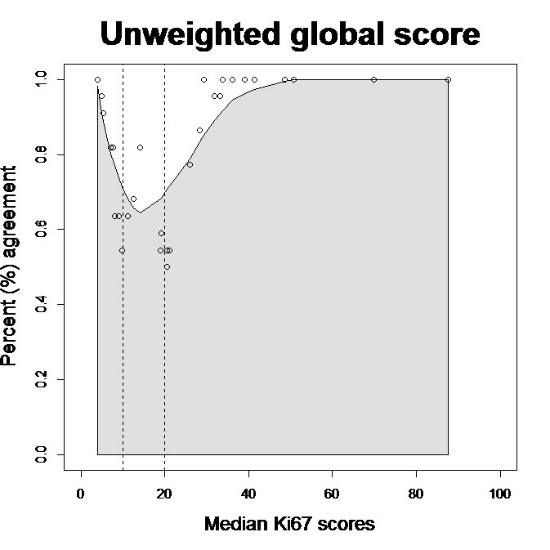3a.** | **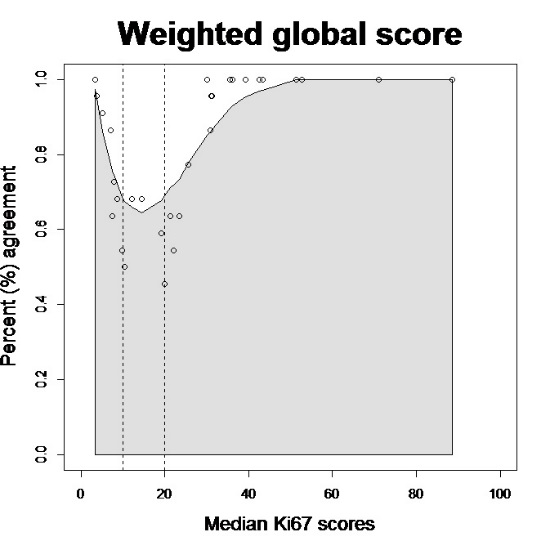3b.** | **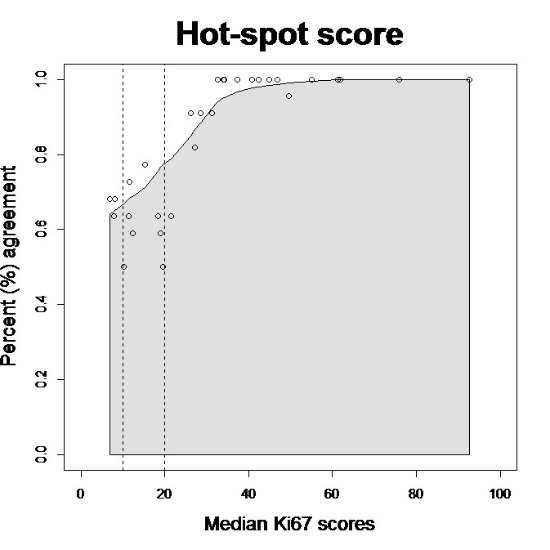3c.** |
| --- | --- | --- |

**Supplemental figure 4.** Variability in Ki67 scores within 10 to 20%. Each line represents Ki67 scores for the same case. Different-colored lines represent data for the 19 (unweighted global – a), 19 (weighted global – b) and 17 (hot-spot – c) cases for which at least one of the represented laboratories reported a score in the range 10%≤ Ki67≤20%.

| 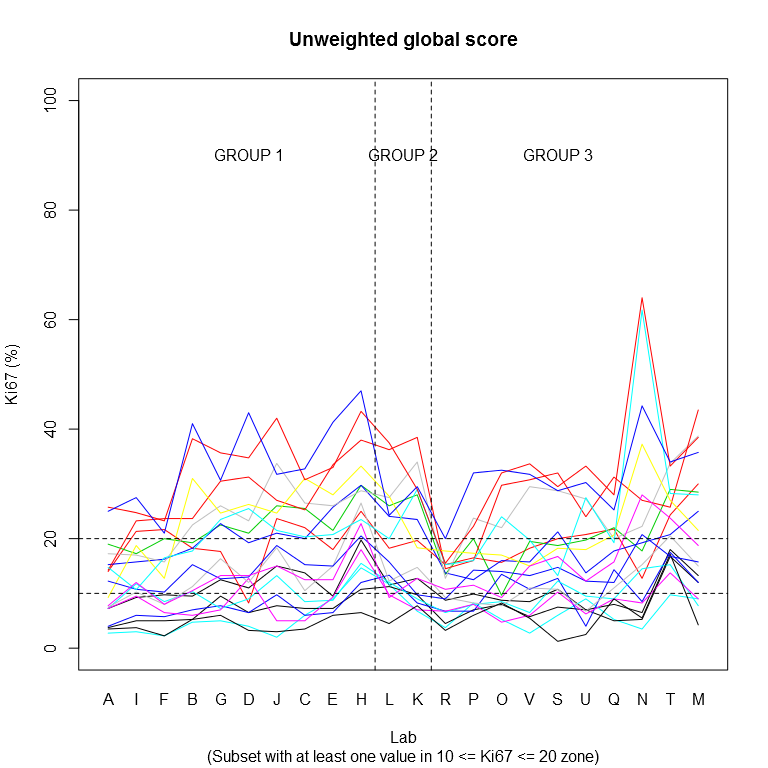  **4a.** | 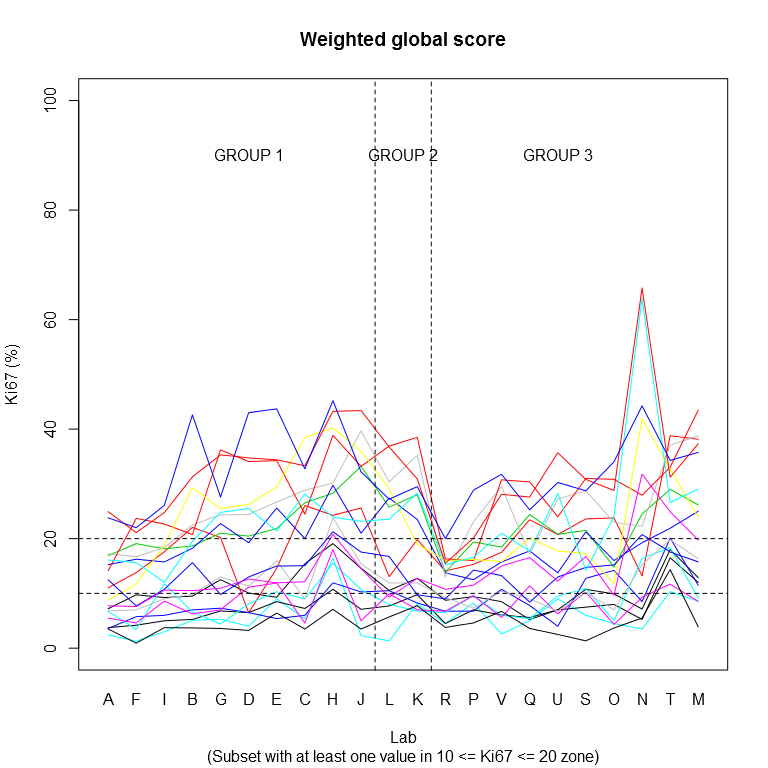  **4b.** |
| --- | --- |
| 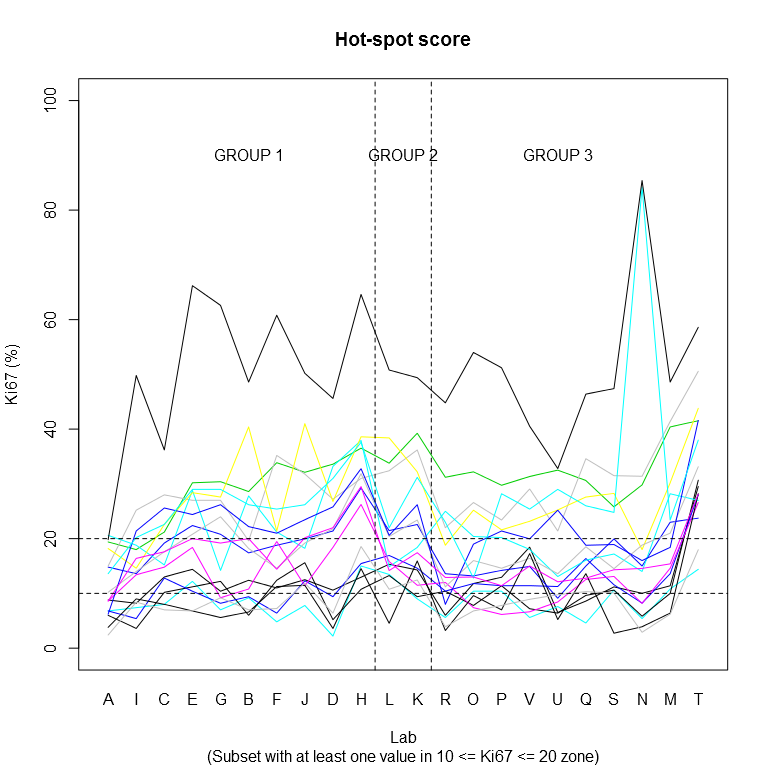  **4c.** |  |

**REFERENCES**

1. Cleveland WS. LOWESS: A program for smoothing scatterplots by robust locally weighted regression. *The American Statistician*. 1981;35:54.

2. R Core Team. R: A language and environment for statistical computing. R foundation for statistical computing, vienna, austria. . 2015.
