# Supplementary material for: Analytical validation of a standardized scoring protocol for Ki67: phase 3 of an international multicenter collaboration
Source: NPJ Breast Cancer. 2016 May 18;2:16014–. doi: 10.1038/npjbcancer.2016.14 (PMC5515324; doi:10.1038/npjbcancer.2016.14)
Supplement: Supplementary Document 2 [file npjbcancer201614-s2.doc]

**Supplemental table 1.** Cohort characteristics (from pathology reports and case notes) of the 30 cases.

| **Age (years)** | | |
| --- | --- | --- |
|  | median / mean | 61 / 65 |
|  | minimum / maximum | 40 / 99 |
| **Grade** | | |
|  | 1 (%) | 3 (10%) |
|  | 2 (%) | 18 (60%) |
|  | 3 (%) | 9 (30%) |
| **Tumor size (mm)** | | |
|  | median / mean | 22 / 27 |
|  | minimum / maximum | 12 / 65 |
| **Number of positive nodes** | | |
|  | 0 (%) | 21 (70%) |
|  | >0 (%) | 9 (30%) |
| **ER (IHC)** | | |
|  | positive | 30 (100%) |

**Supplemental table 2.** Summary statistics for unweighted global scores (0-100%), ordered according to laboratory median.

| **Group 1^1^** | | | | | | | |
| --- | --- | --- | --- | --- | --- | --- | --- |
| Laboratory | min | Q1 | median | mean | Q3 | Max | SD |
| A | 2.5 | 7.3 | 15.0 | 20.9 | 28.0 | 89.2 | 19.4 |
| I | 1.8 | 10.3 | 18.0 | 23.4 | 30.9 | 84.0 | 19.3 |
| F | 2.2 | 8.1 | 18.2 | 22.9 | 26.8 | 90.5 | 20.5 |
| B | 4.8 | 10.4 | 20.9 | 28.2 | 40.3 | 77.5 | 21.5 |
| G | 5.0 | 12.5 | 24.1 | 28.8 | 37.8 | 97.5 | 21.6 |
| D | 3.2 | 11.2 | 24.4 | 27.3 | 40.4 | 96.8 | 21.7 |
| J | 2.0 | 15.0 | 25.3 | 28.0 | 35.4 | 93.5 | 20.1 |
| C | 3.0 | 11.0 | 25.4 | 27.6 | 39.1 | 88.2 | 21.0 |
| E | 4.2 | 10.2 | 25.9 | 29.6 | 42.8 | 92.2 | 21.8 |
| H | 6.0 | 19.9 | 29.8 | 33.1 | 42.9 | 77.0 | 18.6 |
| **Group 2** | | | | | | | |
| Laboratory | min | Q1 | median | mean | Q3 | max | SD |
| L | 4.0 | 12.1 | 25.1 | 26.2 | 35.2 | 82.0 | 17.1 |
| K | 4.0 | 10.5 | 26.1 | 27.8 | 37.7 | 90.2 | 19.7 |
| **Group 3** | | | | | | | |
| Laboratory | min | Q1 | median | mean | Q3 | max | SD |
| R | 3.2 | 8.8 | 14.9 | 21.3 | 29.0 | 86.8 | 20.2 |
| P | 4.2 | 8.7 | 16.9 | 23.1 | 30.4 | 89.0 | 18.9 |
| O | 4.8 | 8.9 | 19.1 | 23.4 | 32.4 | 86.2 | 18.0 |
| V | 1.2 | 10.8 | 19.6 | 24.4 | 31.7 | 83.8 | 18.6 |
| S | 1.2 | 12.4 | 20.6 | 26.8 | 33.3 | 83.8 | 20.5 |
| U | 1.8 | 9.1 | 20.6 | 26.4 | 40.4 | 79.0 | 21.1 |
| Q | 4.8 | 11.2 | 21.2 | 24.6 | 30.9 | 85.2 | 17.6 |
| N | 3.5 | 13.2 | 27.5 | 37.6 | 63.6 | 94.0 | 29.9 |
| T | 4.0 | 17.6 | 27.6 | 30.9 | 41.6 | 79.0 | 16.9 |
| M | 3.8 | 13.7 | 29.2 | 32.8 | 42.6 | 94.8 | 23.1 |
| **Mean^2^** | | | | | | | |
|  | min | Q1 | median | mean | Q3 | max | SD |
|  | 20.9 | 23.7 | 27.0 | 27.1 | 28.6 | 37.6 | 4.10 |

^1^Groups of laboratories are defined by which collection of slides they received for scoring.

^2^Mean for each laboratory computed using scores for 30 slides.

**Supplemental table 3.** **Summary statistics for weighted global scores (0-100%), ordered according to laboratory median.**

| **Group 1^1^** | | | | | | | |
| --- | --- | --- | --- | --- | --- | --- | --- |
| Laboratory | min | Q1 | median | mean | Q3 | Max | SD |
| A | 2.4 | 6.9 | 15.7 | 20.6 | 28.2 | 89.2 | 19.5 |
| F | 0.8 | 7.2 | 16.5 | 23.1 | 33.4 | 90.0 | 22.0 |
| I | 1.8 | 9.6 | 18.3 | 23.2 | 30.6 | 84.0 | 19.3 |
| B | 3.6 | 9.6 | 21.4 | 27.7 | 43.5 | 77.5 | 21.7 |
| G | 3.4 | 10.1 | 24.6 | 28.3 | 37.7 | 97.2 | 22.4 |
| D | 3.2 | 10.3 | 24.9 | 27.5 | 40.8 | 96.8 | 22.0 |
| E | 4.2 | 11.9 | 26.1 | 30.1 | 43.4 | 92.2 | 22.0 |
| C | 1.9 | 9.8 | 27.3 | 28.7 | 41.6 | 88.2 | 21.2 |
| H | 5.5 | 19.5 | 29.9 | 34.9 | 46.2 | 86.1 | 20.4 |
| J | 2.0 | 14.6 | 32.7 | 30.4 | 43.9 | 93.5 | 21.4 |
| **Group 2** | | | | | | | |
| Laboratory | min | Q1 | median | mean | Q3 | max | SD |
| L | 1.3 | 10.5 | 27.3 | 26.6 | 36.9 | 82.0 | 18.2 |
| K | 4.0 | 10.3 | 28.2 | 28.1 | 39.5 | 90.2 | 19.9 |
| **Group 3** | | | | | | | |
| Laboratory | min | Q1 | median | mean | Q3 | max | SD |
| R | 3.5 | 8.8 | 14.7 | 21.4 | 29.0 | 86.8 | 20.2 |
| P | 4.2 | 9.6 | 16.4 | 22.9 | 28.7 | 89.0 | 19.2 |
| V | 0.8 | 10.5 | 19.7 | 24.0 | 31.3 | 83.8 | 18.4 |
| Q | 2.3 | 8.6 | 20.2 | 23.9 | 30.2 | 89.3 | 19.1 |
| U | 1.5 | 9.1 | 20.8 | 26.7 | 40.4 | 79.0 | 21.3 |
| S | 1.3 | 11.4 | 22.6 | 27.2 | 33.7 | 83.8 | 20.6 |
| O | 3.7 | 9.7 | 23.4 | 24.6 | 33.6 | 86.2 | 19.3 |
| N | 3.5 | 13.7 | 29.9 | 38.6 | 64.2 | 94.0 | 29.8 |
| T | 3.7 | 18.7 | 30.1 | 32.4 | 41.6 | 79.0 | 17.7 |
| M | 1.9 | 13.6 | 31.2 | 33.1 | 42.6 | 94.8 | 23.1 |
| **Mean^2^** | | | | | | | |
|  | min | Q1 | median | mean | Q3 | max | SD |
|  | 20.6 | 23.9 | 27.4 | 27.4 | 29.8 | 38.6 | 4.5 |

^1^Groups of laboratories are defined by which collection of slides they received for scoring.

^2^Mean for each laboratory computed using scores for 30 slides.

**Supplemental table 4.** Summary statistics for hot-spot scores (0-100%), ordered according to laboratory median.

| **Group 1^1^** | | | | | | | |
| --- | --- | --- | --- | --- | --- | --- | --- |
| Laboratory | min | Q1 | median | mean | Q3 | Max | SD |
| A | 2.4 | 8.7 | 19.6 | 24.4 | 30.1 | 95.0 | 21.7 |
| I | 3.6 | 13.7 | 23.3 | 28.6 | 40.3 | 93.6 | 21.1 |
| C | 7.0 | 15.8 | 26.8 | 32.6 | 44.1 | 92.6 | 22.1 |
| E | 6.8 | 20.2 | 29.6 | 35.8 | 49.1 | 97.8 | 22.5 |
| G | 5.6 | 15.4 | 29.7 | 35.6 | 50.4 | 99.0 | 24.8 |
| B | 6.0 | 17.6 | 29.7 | 34.4 | 48.0 | 98.0 | 23.2 |
| F | 4.8 | 15.7 | 30.2 | 33.6 | 43.2 | 98.6 | 23.6 |
| J | 7.8 | 18.5 | 32.0 | 35.2 | 49.5 | 89.6 | 21.2 |
| D | 2.2 | 21.5 | 32.1 | 35.8 | 52.5 | 97.6 | 23.9 |
| H | 10.8 | 29.3 | 37.8 | 41.6 | 54.4 | 90.9 | 20.6 |
| **Group 2** | | | | | | | |
| Laboratory | min | Q1 | median | mean | Q3 | Max | SD |
| L | 4.5 | 16.1 | 36.0 | 32.9 | 42.8 | 83.6 | 18.3 |
| K | 9.0 | 17.7 | 37.5 | 35.8 | 48.2 | 93.0 | 20.5 |
| **Group 3** | | | | | | | |
| Laboratory | min | Q1 | median | mean | Q3 | Max | SD |
| R | 3.2 | 11.4 | 24.1 | 28.8 | 41.6 | 87.8 | 21.6 |
| O | 6.8 | 12.8 | 24.8 | 30.0 | 42.6 | 86.8 | 20.7 |
| P | 6.2 | 13.3 | 25.3 | 30.6 | 41.6 | 90.8 | 21.4 |
| V | 5.6 | 16.6 | 25.9 | 29.9 | 37.1 | 90.8 | 19.9 |
| U | 5.2 | 11.5 | 27.5 | 31.2 | 39.9 | 94.2 | 23.6 |
| Q | 4.6 | 15.3 | 29.1 | 31.2 | 42.2 | 92.6 | 20.2 |
| S | 2.7 | 14.4 | 29.9 | 31.6 | 37.9 | 90.5 | 21.8 |
| N | 2.9 | 14.1 | 30.1 | 38.4 | 76.0 | 91.4 | 31.2 |
| M | 6.2 | 16.1 | 34.2 | 37.2 | 50.6 | 95.0 | 24.4 |
| T | 14.4 | 28.5 | 42.4 | 43.6 | 51.0 | 85.8 | 17.5 |
| **Mean^2^** | | | | | | | |
|  | min | Q1 | median | mean | Q3 | Max | SD |
|  | 24.4 | 30.7 | 33.3 | 33.6 | 35.8 | 43.6 | 4.4 |

^1^Groups of laboratories are defined by which collection of slides they received for scoring.

^2^ Mean for each laboratory computed using scores for 30 slides.

**Supplemental table 5.** Variance components estimates and corresponding credible intervals.

| **Component** | **Scoring method** | **Variance estimate (95% credible intervals)** |
| --- | --- | --- |
| Biological | Unweighted global | 1.46 (0.78─2.26) |
|  | Weighted global | 1.66 (0.90─2.61) |
|  | Hot-spot | 1.11 (0.60─1.76) |
| Residual | Unweighted global | 0.13 (0.12─0.14) |
|  | Weighted global | 0.15 (0.13─0.17) |
|  | Hot-spot | 0.13 (0.12─0.14) |
| Laboratory | Unweighted global | 0.06 (0.03─0.11) |
|  | Weighted global | 0.09 (0.04─0.15) |
|  | Hot-spot | 0.06 (0.03─0.11) |
| Section | Unweighted global | 2x10^-3^ (1x10^-16^─0.01) |
|  | Weighted global | 2x10^-3^ (2x10^-16^─9x10^-3^) |
|  | Hot-spot | 6x10^-4^ ( 2x10^-16^─5x10^-3^) |
