# Supplementary material for: Analytical validation of a standardized scoring protocol for Ki67: phase 3 of an international multicenter collaboration
Source: NPJ Breast Cancer. 2016 May 18;2:16014–. doi: 10.1038/npjbcancer.2016.14 (PMC5515324; doi:10.1038/npjbcancer.2016.14)
Supplement: Supplementary Tables [file npjbcancer201614-s3.pdf]

Instructions for Ki67 Reproducibility Study Phase 3: Core Biopsies

Version: July 17, 2014

NOTE: It may be easier you for you to read these instructions in their entirety first. Although they appear long, much space is taken up by graphics.

NOTE: Please do <u>not</u> use Safari web browser on a Mac.

NOTE: Please do <u>not</u> use Chrome (on either PC or Mac).

NOTE: The website software works best with Mozilla Firefox browser, especially in a Windows environment.

NOTE: The same scorer should score all core biopsies, using both the hotspot method and representative fields method (as described below).

Please do not allow any other scorer to score the biopsies unless they have also completed the calibration exercise, are willing to independently score all cases using both scoring methods, and have registered with Rebecca Enos and Sam Leung their intent to be part of the formal scoring exercise.

The following instructions describe a standardized procedure for Ki67 scoring on core-needle biopsy specimens.

Both the 1) hotspot and 2) average Ki67 score will be captured for each specimen.

Thirty (30) core biopsies will be assessed in this manner by each participant.

Key materials and equipment for scoring:

- A Bright Field Microscope, a range of objectives, a 10x ocular eyepiece

    The objectives can include 4x, 10x, 20x and 40x. The resulting magnification would then be 40x, 100x, 200x and 400x.

    A High Power Magnification (HPM) is derived by multiplying 10x ocular and 40x objective = 400x.

- Either a slide micrometer or clear ruler (for measuring microscope field diameter)
- Adjacent computer with internet connection, and sound/speakers activated
- **Appendix 1, at the end of this document:** *"What counts as 'positive' Ki67 staining?"* This Appendix describes and illustrates the staining intensities that should be considered positive/negative (same as used in the calibration exercise).

Contact if you have questions:

Samuel Leung at Samuel.Leung@vch.ca or Phone: 1-604-875-4111 x62649

You can also click the "Troubleshooting" button at the top of each screen for help. 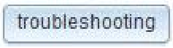

Before logging onto the scoring website:

1) Please make sure that the Java runtime environment is up to date on your computer. You can verify this at the following website:

   http://www.java.com/en/download/installed.jsp

2) Please also take the following steps:

   a) In the blank box above the Windows Start button on your computer, type “Configure Java” and press Enter.

   b) In the Java Control Panel that appears, click “Settings” under “Temporary Internet Files”.

   c) Uncheck “Keep temporary files on my computer”.

   d) Click “Delete files...”

   e) Place a check in all three checkboxes that appear in the next screen.

   f) Click OK on each screen to leave the Java Control Panel.

How to respond to website warnings/messages:

If you encounter a “Security Warning” that asks *“Do you want to view only the webpage content that was delivered securely?”*, click “No”.

If you encounter *“Activate Java(TM) Platform SE 7 U,”* click 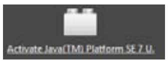 to activate.

If you encounter a *“Do you want to run this application?”* message with this icon 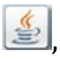, check the “Do not show this again for apps....” box, then click “Run”.

Other set-up notes:

If you use Windows 8, the website only works in “Desktop mode”. There have been issues reported with the Windows 8 system with Kaspersky anti-virus software installed; the following website offers a workaround solution if you have that issue: http://support.kaspersky.com/9931

Firefox users:

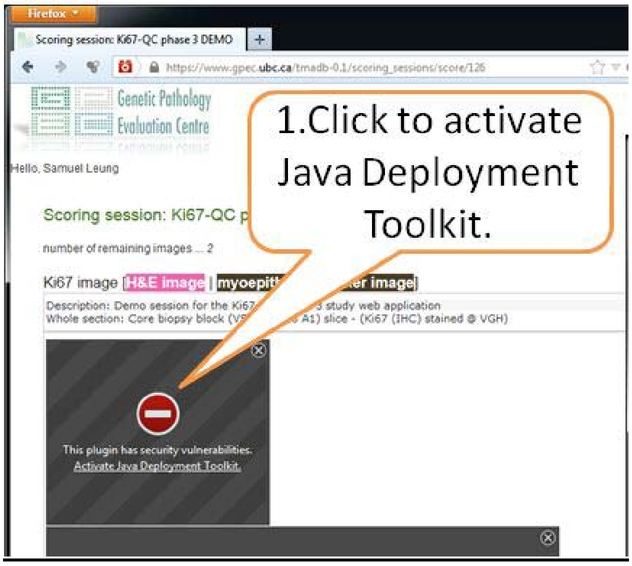

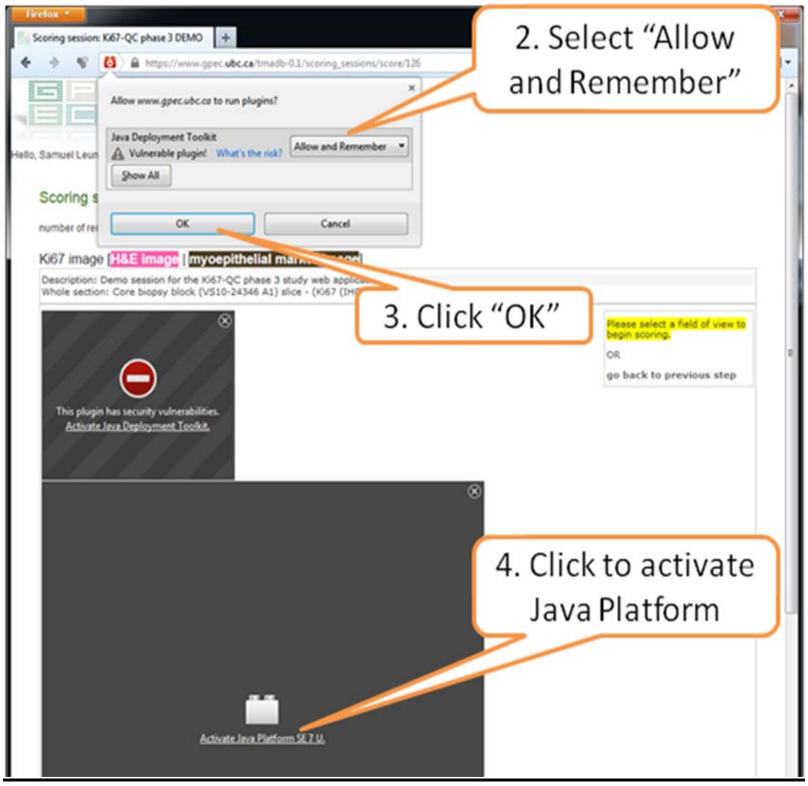

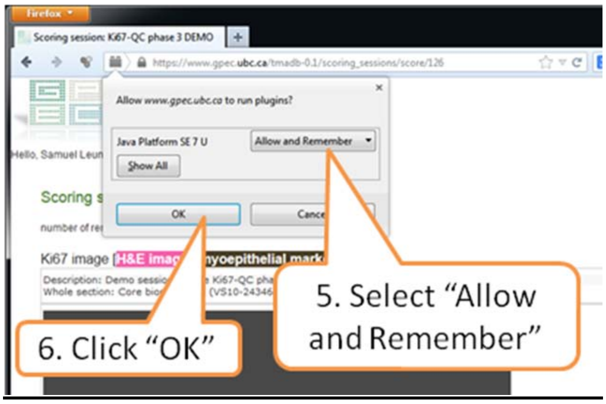

➔ **Before you begin, please watch the brief training video at the link below:**
http://www.youtube.com/playlist?list=PLd730BvCyxMWaOZgpghgTFbzC5uWe9uhi

Instructions for the practice exercise

After watching the brief training video, you will then practice using the web application by scoring two "beta" cases:

1. Connect to the scoring website at http://www.gpec.ubc.ca:8080/tmadb-0.1/ **(Figure 1)** and sign in using the username and password sent to you by Rebecca Enos.

Figure 1. Sign-in screen

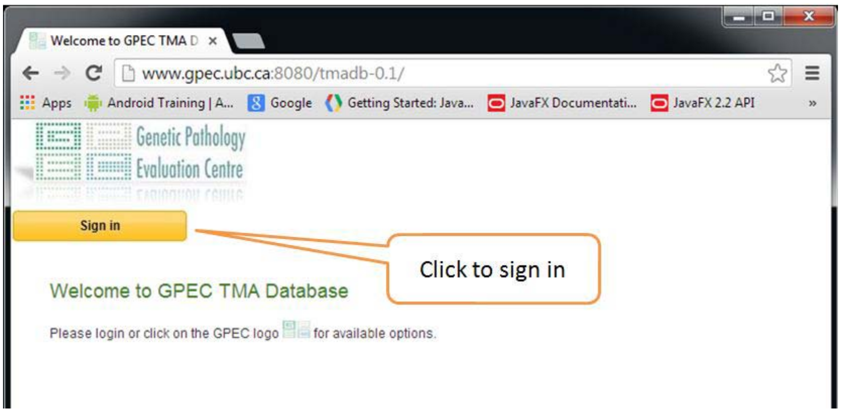

2. In the "Welcome to scoring session" screen that appears **(Figure 2)**, click on **"Ki67-QC phase 3 beta test"**.

Figure 2. Selecting your scoring session

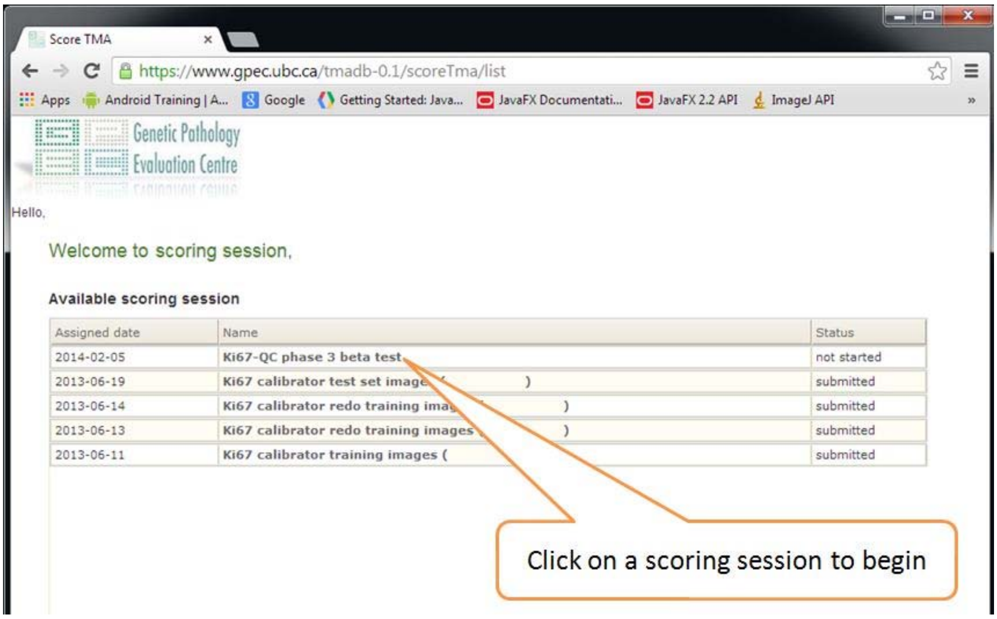

3. Click "OK" when you receive the following prompt. (There is no actual glass slide for this practice session. Instead, you will use a virtual slide for practicing.)

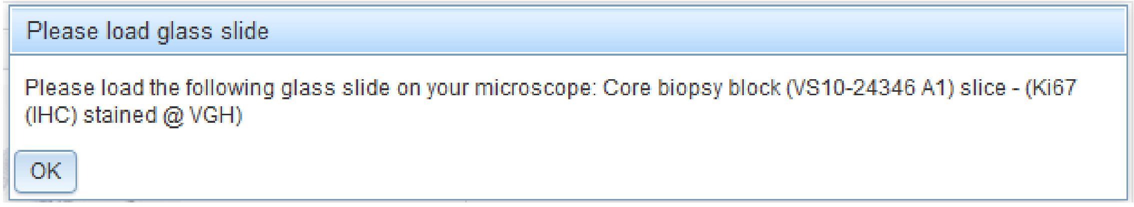

4. To call up the virtual slide for the first "beta" case, click on the following link. At the prompt, enter the same username and password you used earlier.

   First beta case: Patient VS10-24346 A1:
   http://www.gpec.ubc.ca:8080/tmadb-0.1/whole_section_slices/show/4

   The virtual slide should open in a **separate browser window** so that you can easily consult both windows.

5. Thus, **in the first window** ("Scoring session" tab), you will see a low-resolution image. You can zoom by clicking the + or – buttons, or by using your mouse's scroll wheel. The thumbnail (smaller picture) beside the image helps you locate where you are in the core biopsy. If you wish to hide it, you can click "hide thumbnail".

   In the second window ("Whole section slice" tab), you will see a high-resolution Aperio 20x image, which is your "virtual slide" and permits you to zoom in to see detail. You can zoom in by clicking on that image.

   In this practice session, you will consult this high-resolution Aperio 20x image for choosing your fields to score. (In the actual session, you will use glass slides.)

6. Now, please follow the "**Scoring instructions for the actual "phase 3" exercise"**, which begin on the next page, pretending that the Aperio 20x high-resolution image is an actual glass slide.

   NOTE: For scoring during this practice session, you do not actually have to count hundreds of nuclei per core. Please do, however, count enough cells to get used to the nuclei counter web application.

   NOTE: Please also note there are no myoepithelial marker images for the practice cases. There are such images for the actual cases.

7. When you are done with the first beta case, you will receive a prompt similar to the one shown in step #3 above. Follow the same steps as above, this time for this second "beta" case. The virtual slide for this second "beta" case is found at the link below. Use the same username and password you used earlier:

   Second beta case: Patient VS13-29105 A1:
   http://www.gpec.ubc.ca:8080/tmadb-0.1/whole_section_slices/show/6

8. You are now done with the practice session. Please now go to **"Scoring instructions for the actual "phase 3" exercise"**, below, to begin the actual scoring on glass slides.

Scoring instructions for the actual "phase 3" exercise

1. First, look closely at **Appendix 1** at the end of this document (*"What counts as 'positive' Ki67 staining?"*).

   Appendix 1 contains visual examples demonstrating the intensities of Ki67 staining that should be considered "positive" or "negative".

   NOTE: <u>Any</u> amount of brown nuclear staining should be considered positive.

2. If you would like to go directly from the practice session to the actual scoring exercise, click on the **"Genetic Pathology Evaluation Centre" logo** in the upper left corner of the browser, then click **"Home" (Figure 3)**. (You can do this any time you wish to return to the Welcome page.)

Figure 3. Returning to the Welcome page

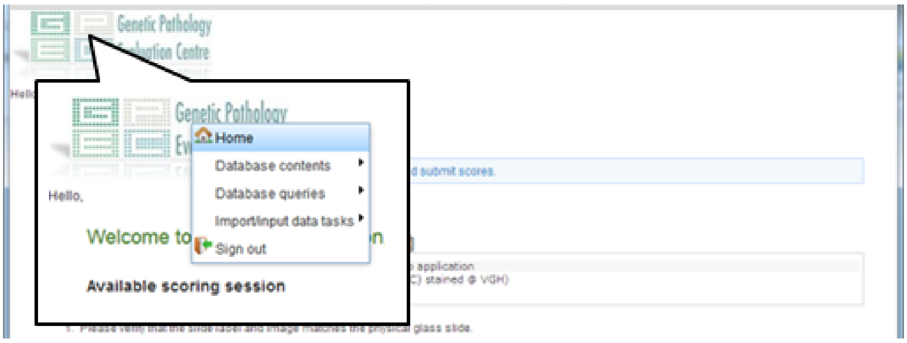

Or, if you have signed out, you can return to the scoring website by:

- Going to http://www.gpec.ubc.ca:8080/tmadb-0.1/
- Signing in (same username and password as in the practice session).
- Clicking on the **"Genetic Pathology Evaluation Centre" logo**.
- Clicking **"Home"**.

3. In the "Welcome to scoring session" screen that appears **(Figure 4)**, click on **"Ki67 phase 3 – nuclei count on core biopsy"**.

Figure 4. Selecting your scoring session

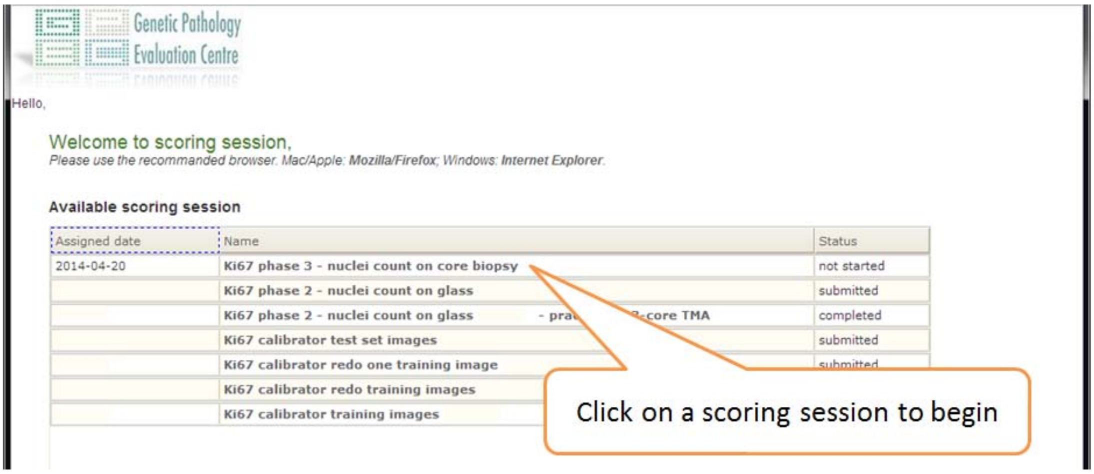

4. The next screen that appears will show a low-power view of the Ki67-stained slide you are going to score **(Figure 5)**.

5. Click the "Slide label" tab. **Confirm that the label on the digital Ki67 image matches the label on the Ki67-stained core on your glass slide.**

   If they do not match, please contact Samuel Leung at Samuel.Leung@vch.ca or Phone: 1-604-875-4111 x62649.

   The Slide View tab, which provides a macroscopic image of the slide, can also help with verification.

Figure 5. Web screen with low-power image of core biopsy, and where you note the 40x field diameter of your microscope (an example diameter is shown below)

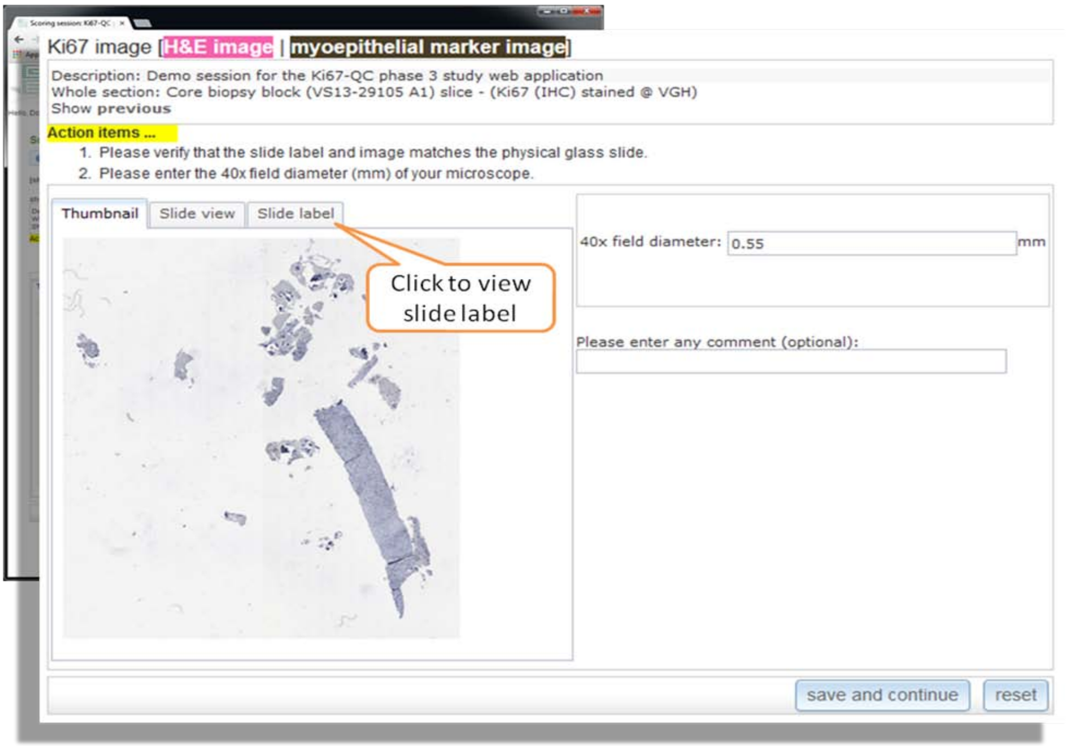

6. Enter the **40x field diameter** of your microscope **(Figure 5)**.

   If you do not have a slide micrometer to measure the 40x field diameter of your microscope, you can easily use a clear ruler instead to measure your low-power field diameter, and then calculate your 40x High-Power Field diameter by following the steps below:

   <u>Determining the size of your High-Power Field (HPF) with a clear ruler</u>:

   First, use a clear plastic ruler to measure the microscopic field diameter of your *low*-power field. Place the ruler on the stage as if it were a slide, and look through your ocular lenses.

   Then calculate the 40x high-power field diameter as follows:

$$\frac{\textbf{(Low-Power Objective Magnification x Microscopic Field Diameter)}}{\textbf{40}} = \textbf{40x field diameter}$$

Example of calculating 40x field diameter:

At 2.5x, the field of view diameter is measured with the clear plastic ruler to be 8.8 mm. Plugging into the formula gives the following 40x field diameter:

$$\frac{(2.5x) \text{ x } (8.8mm)}{40x} = 0.55 \text{ mm}$$

NOTE: If you are unable to determine the HPF diameter, the computer will default to 0.6 mm.

7. Click "**save and continue**".

8. Selection and scoring of the hotspot

SELECTING THE HOTSPOT:

The next screen **(Figure 6)** will ask you to choose a hotspot on the **glass slide**, and to indicate its location on the digital image.

You will always be asked to select a hotspot field, even in homogenous cases.

Figure 6. Selecting the hotspot

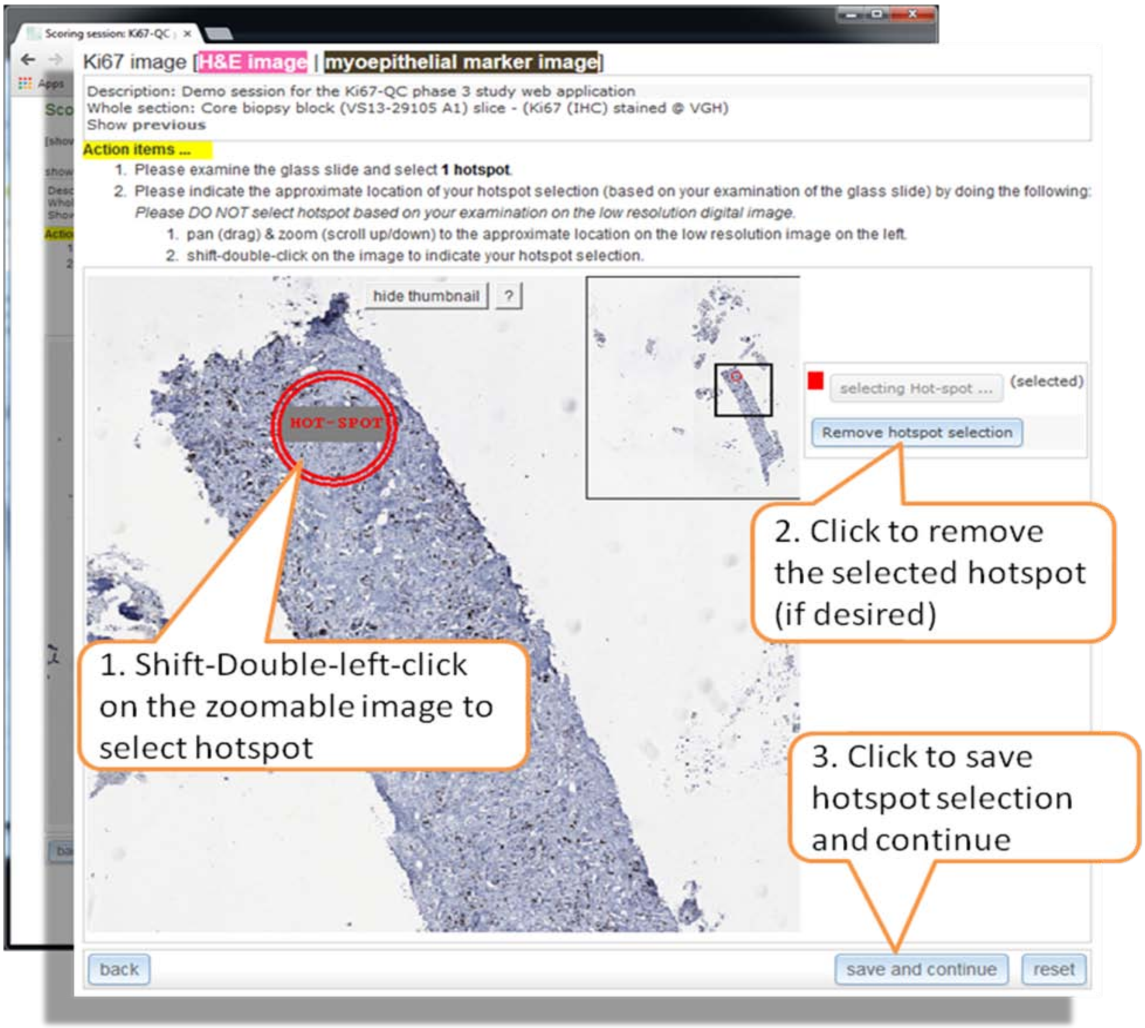

a) Examine the entire physical **glass slide** using low-power magnification (with 4x, 10x objectives).

   Identify the area you consider to represent <u>the highest Ki67</u> on the **glass slide**.

   If you wish to view images of the H&E and myoepithelial marker-stained slides, click the "H&E image" and "myoepithelial marker image" links at the top of the browser window. In those images, you can zoom/magnify.

> **IMPORTANT:** Please do not select the hotspot based on your examination of the digital image. Rather, base your selection on your examination of the <u>actual glass slide</u>.

b) Then, go back to the digital image on the website **(Figure 6)**. You will see the "Hotspot" button reads "selecting Hotspot".

> **<u>Notes on marking the digital image</u>:**
>
> - You can move the image by dragging it.
> - You can zoom by using your mouse's scroll wheel. Since the digital image is low resolution, you can only zoom to a certain extent.
> - You can preview where the field will be marked by pressing Shift and single-clicking.
> - The website will also give you options to remove your selections ("Remove hotspot selection" or "reset").

   On the area in the digital image that corresponds with the hotspot you identified on the glass slide, **double-click** while holding down the **Shift key**.

   This will mark the area with a **red circle** matching the 40x field diameter of your microscope. This area will represent the hotspot.

      Red circle = Hotspot *(NOTE: Circle will also be labeled "Hotspot".)*

c) Click **"save and continue"**. A message will appear: "ok to save hotspot selection?" Press **"OK"** to continue.

SCORING THE HOTSPOT:

Make sure your computer's sound is turned on.

d) In the next screen that appears **(Figure 7, next page)**, click inside the HOTSPOT circle (red circle). This will activate the nuclei counter web application **(Figure 7, next page)**.

Figure 7. Clicking on the circle (top figure) will activate the nuclei counter (bottom figure)

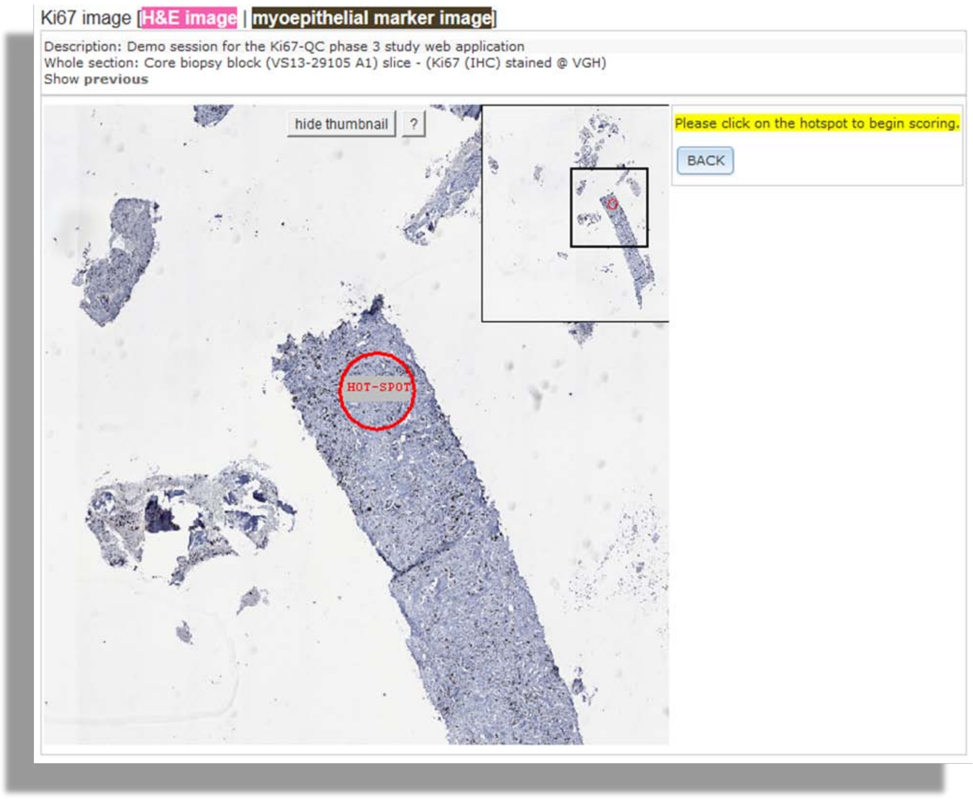

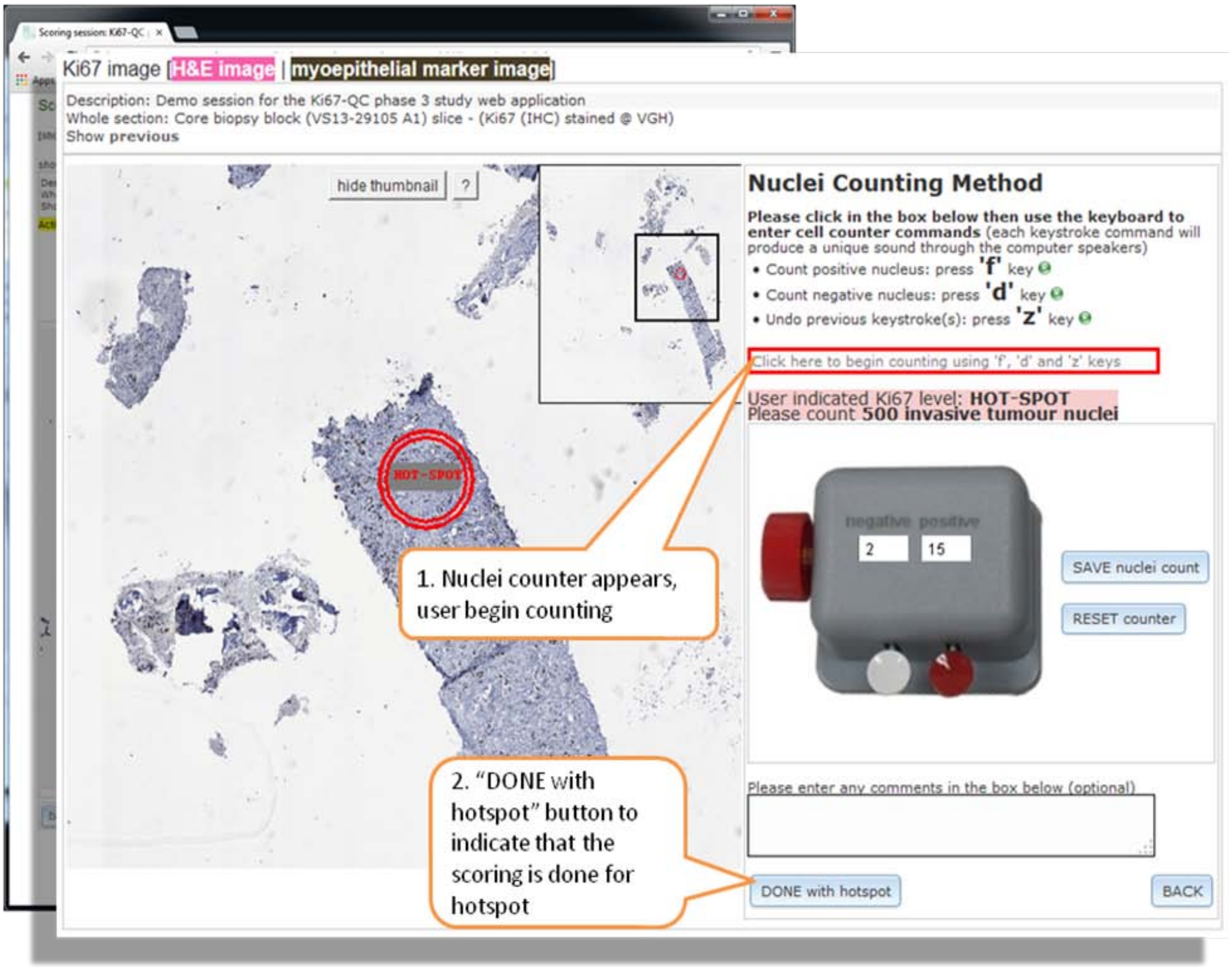

e) Go back to your **glass slide**. Position your high-powered (40x objective) microscope field to the area on the actual core biopsy glass slide that corresponds with the HOTSPOT circle on the digital image.

f) Start scoring the glass slide at the top of the hotspot field of view matching the highlighted circle on the digital image. Count nuclei in a "typewriter" pattern from the top, until **either** 500 invasive tumour nuclei in total are scored **or** the entire field of view has been scored, whichever comes first.

Please remember to use the nuclei counter application as you score the glass slide. This records your scoring data.

You do this by placing your cursor inside the red box labelled **"Click here to begin counting using 'f', 'd' and 'z' keys" (Figure 7, above)**, and then pressing the following keys as appropriate:

Press the **"f"** key for a positive nucleus.

Press the **"d"** key for a negative nucleus.

Press the **"z"** key to undo a score.

Each of these keystrokes will produce a different sound.

Do not click outside the red box.

You will see your counts appearing in the picture of the counter on the web screen.

**Reminder:** Please score invasive tumour nuclei.

**Saving your work:**<br>Make sure to click "SAVE nuclei count" periodically.<br>If you do not save after three hours, you will lose your work. A message will warn you 1 minute before the time-out.<br>Saving the nuclei count of the hotspot may take up to a minute.

If, as you count, you wish to re-do your counting of the field, click "RESET counter".

If you have any comments, please put them in the comment field.

When you reach 500 nuclei, the software will make a dinging sound, and a message will appear:

"500 nuclei have been counted. Please click on the 'DONE with hotspot' button to indicate that you have finished scoring the hotspot.".

g) If you are done scoring the hotspot, click **"DONE with hotspot"**.

9. Selection and scoring of representative fields

Now you will select and score representative fields.

In the next screen that appears **(Figure 8)**, you will specify the percentages of invasive tumour in the glass slide that exhibit various levels of Ki67 scores ("levels" is defined below, in step 9b).

SPECIFYING THE PERCENTAGES

a) Examine the entire **glass slide** section using low-power magnification (with 4x, 10x objectives).

   If you wish to view images of the H&E and myoepithelial marker-stained slides, click the "H&E image" and "myoepithelial marker image" links at the top of the browser window **(Figure 8)**. In those images, you can zoom/magnify.

b) **Estimate the percentages** of the invasive tumour in the **glass slide** that exhibit the following Ki67 levels:

   - Negative i.e., contains invasive cells but a very low (including zero) percentage of positive invasive cells
   - Low
   - Medium
   - High

Figure 8. Screen where you fill in the percentages

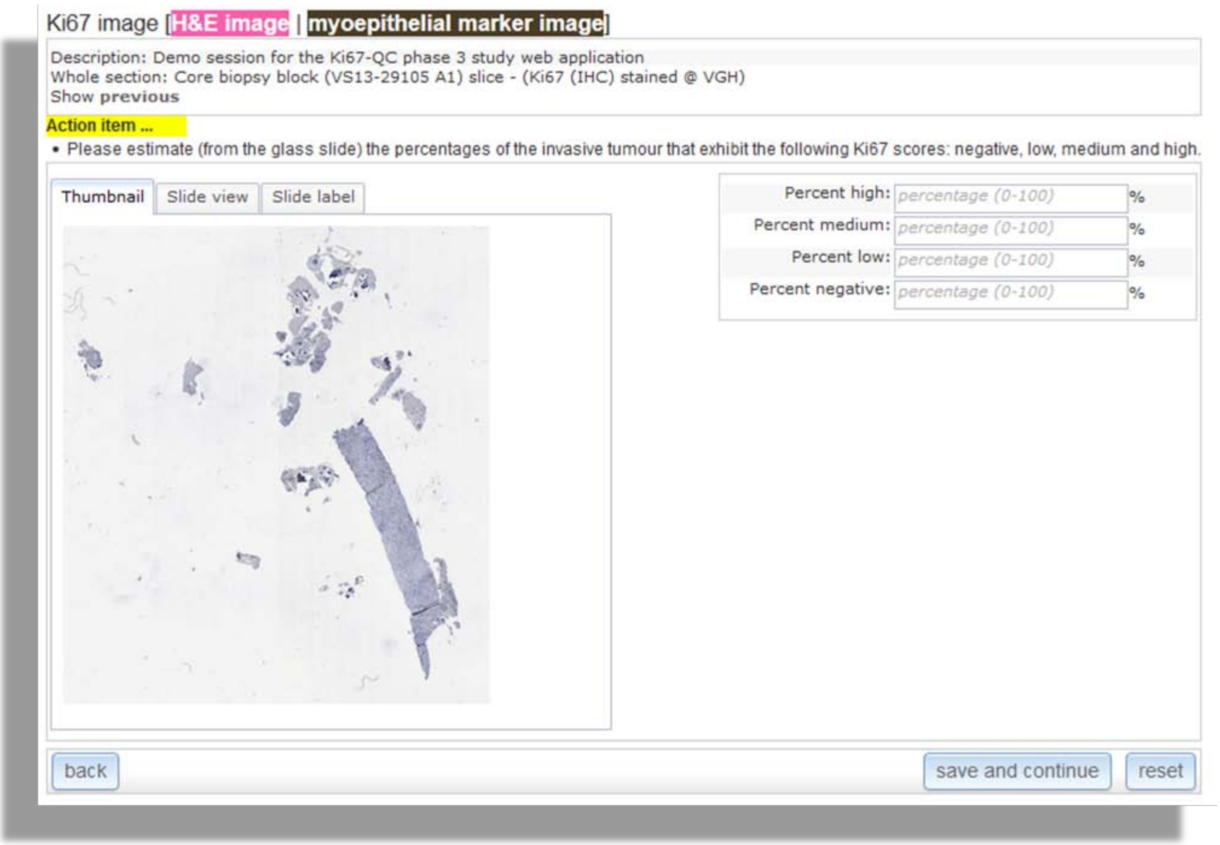

If staining is homogeneous across all invasive tumour (i.e., equal Ki67 across the entire invasive tumour component), you can enter 100% in the appropriate Ki67 level, and 0% for the other Ki67 levels.

Areas with increased Ki67 positivity, including areas towards the edge of the tumour, as well as non-proliferating areas of invasive tumour cells, must be included in your assessment.

IMPORTANT: Heterogeneity of percentage of cells staining positive frequently occurs across a section.

Therefore, scorers should select regions for scoring that are High, Medium, Low, or Negative in relation to the overall percentage positivity.

Thus, "Negative, "Low", "Medium", and "High" are meant to be relative determinations, based on each particular case, and do not reflect specific absolute values.

Please see the examples provided below, which illustrate this concept.

Examples illustrating how to estimate the percentage of Ki67-stained invasive tumour nuclei:

Example 1

You will assign percentages based on the invasive component. Therefore, if the entire core looks like this...

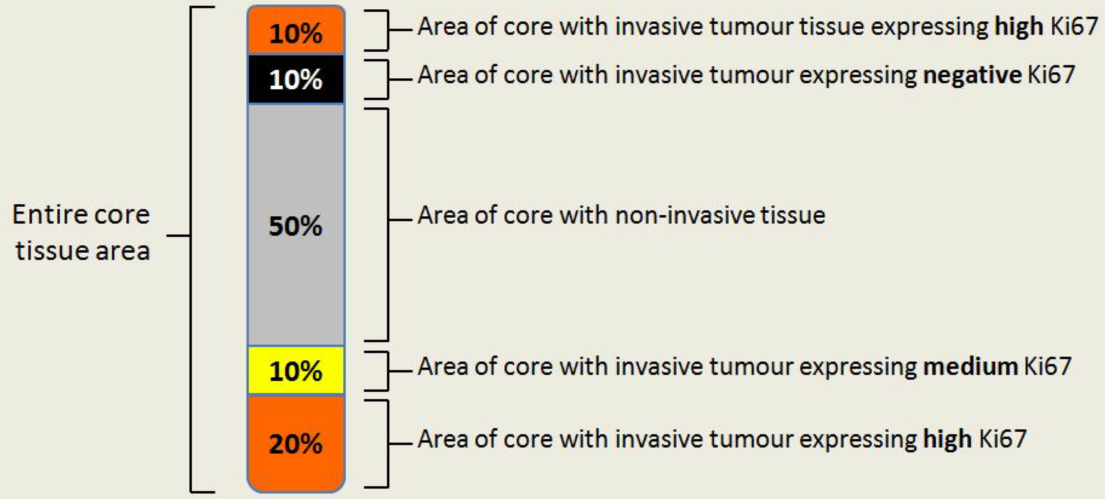

...This means 50% of the core is invasive. So, you would assign percentage of invasive tumour to a Ki67 category as follows:

Ki67 category	Percentage of invasive tumour
Negative	10%/50% = **20%**
Low	0%/50% = **0%**
Medium	10%/50% = **20%**
High	(10% + 20%)/50% = **60%**

Example 2

You will assign percentages based on the <u>invasive</u> component. Therefore, if the entire core looks like this...

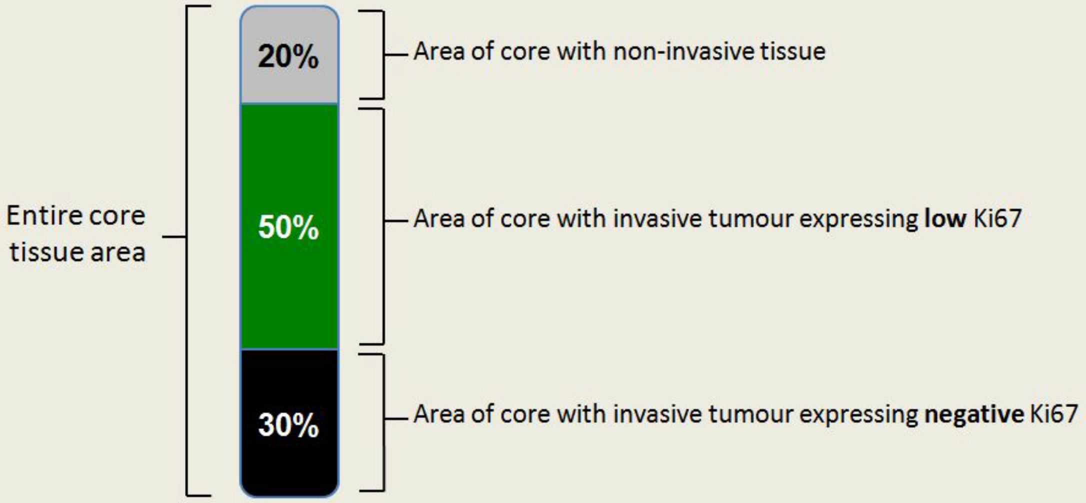

...This means <u>80%</u> of the core is <u>invasive</u>. So, you would assign percentage of invasive tumour to a Ki67 category as follows:

Ki67 category	Percentage of invasive tumour
Negative	30%/80% = **37.5%**
Low	50%/80% = **62.5%**
Medium	0%/80% = **0%**
High	0%/80% = **0%**

Example 3

You will assign percentages based on the <u>invasive</u> component. Therefore, if the entire core looks like this...

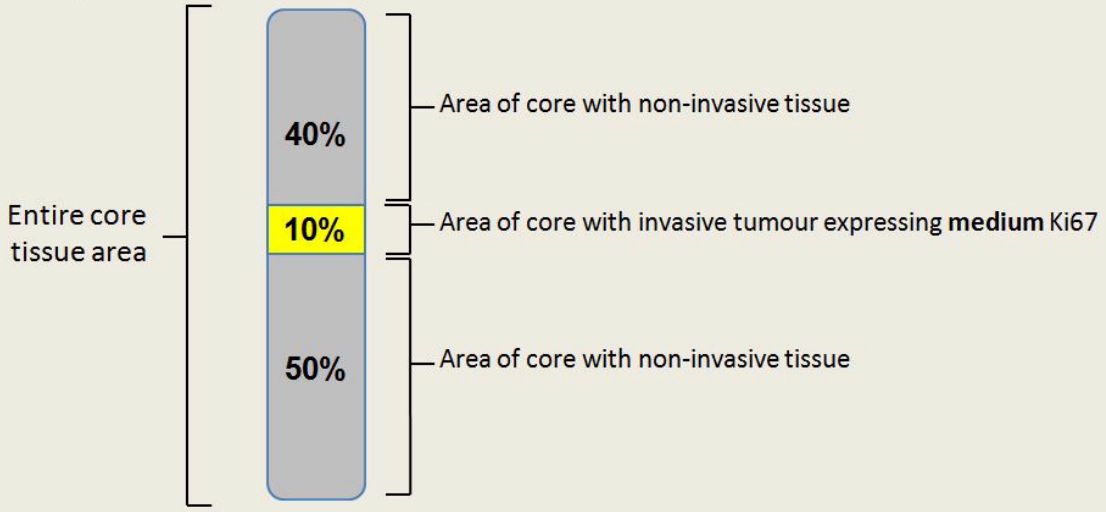

...This means <u>10%</u> of the core is <u>invasive</u>. So, you would assign percentage of invasive tumour to a Ki67 category as follows:

Ki67 category	Percentage of invasive tumour
Negative	0%/10% = **0%**
Low	0%/10% = **0%**
Medium	10%/10% = **100%**
High	0%/10% = **0%**

c) Return to your computer screen.

   Now, **fill in your percentages into the boxes** labeled “Percent high”, “Percent medium,” “Percent low”, and “Percent negative”, which appear beside the image of the core biopsy **(Figure 8, above)**.

d) If you have any comments on the slide, please enter them into the comment field.

e) Click **“save and continue**”.

   A box will appear that asks, “ok to save percentages of Ki67 scores?”

   If you are ready to save your percentages, click **“OK”**. If you are not ready, you can click “Cancel”.

SELECTING THE REPRESENTATIVE FIELDS

In the next screen that appears **(Figure 9)**, you will click on the regions in the digital image that you believe are representative of the Ki67 levels you indicated.

Figure 9. Selecting the representative fields

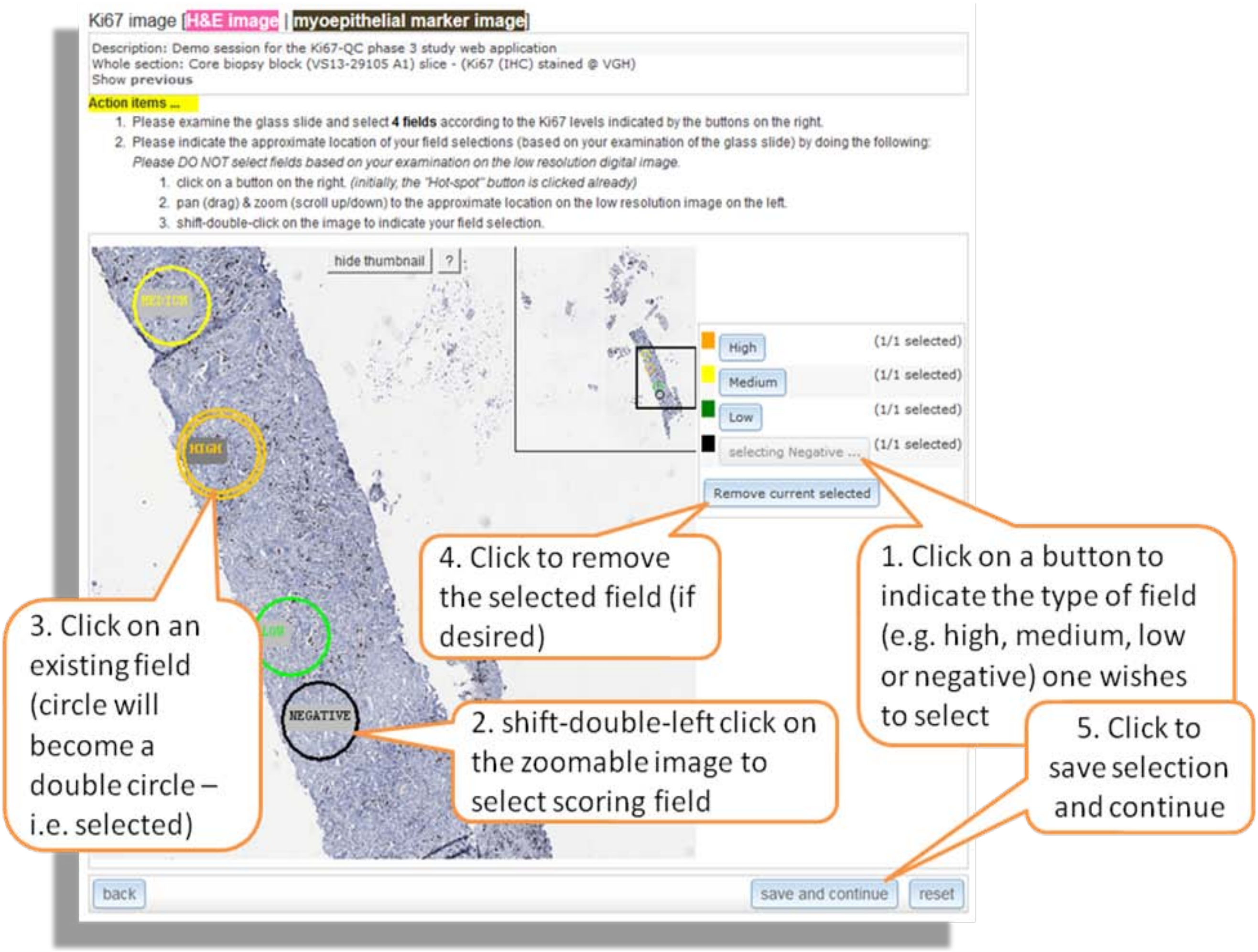

f) First, examine the entire physical **glass slide** to choose the fields you believe are representative of the Ki67 levels you indicated.

g) Then, on the web screen, click on the button indicating the Ki67 level of the field you want to select (i.e., "High", "Medium", "Low", or "Negative") **(Figure 9, above)**.

   To indicate the location of the field, **double-click** on that area in the digital image while holding down the **Shift key** .

   These areas will be marked with colour circles, as follows:

   Orange circle = High
   Yellow circle = Medium
   Green circle = Low
   Black circle = Negative (NOTE: Negative fields will also be scored, even if they contain zero or very few positive cells)
   (NOTE: Circles will also be labeled accordingly.)

   The computer will show you how many fields per Ki67 level you will need to select. (The computer determines these numbers based on a programmed algorithm, explained in **Appendix 2**.)

   For example, if your percentage for "High" cues the computer to assign 2 fields to "High", and you have so far specified the location of 1 of those 2 fields, then "1/2 selected" will show beside the "High" button.

h) When you are done selecting your fields, click **"save and continue"**.

i) A box will appear that asks, "ok to save field selections?" If you are ready to save your field selections, click "**OK**". If you are not ready, you can click "Cancel".

SCORING THE REPRESENATIVE FIELDS

Make sure your computer's sound is turned on.

j) In the next screen that appears, click inside one of the circles. This will activate the nuclei counter web application **(Figure 10, next page)**.

k) Go back to the actual core biopsy **glass slide**. Position your high-powered (40x objective) microscope field to the area on the glass slide that corresponds with the circle you just clicked.

l) Start scoring **the glass slide** at the top of the field of view matching the highlighted circle on the digital image. Count nuclei in a "typewriter" pattern from the top, until **either** 100 invasive tumour nuclei in total are scored **or** the entire field of view has been scored, whichever comes first.

Please remember to use the nuclei counter application as you score the glass slide. This records your scoring data.

You do this by placing your cursor inside the red box labelled **"Click here to begin counting using 'f', 'd' and 'z' keys" (Figure 10)**, and then pressing the following keys as appropriate:

Press the **"f"** key for a <u>positive</u> nucleus.

Press the **"d"** key for a <u>negative</u> nucleus.

Press the **"z"** key to undo a score.

Each of these keystrokes will produce a different sound.

Do not click outside the red box.

You will see your counts appearing in the picture of the counter on the web screen.

Figure 10. Scoring the representative fields

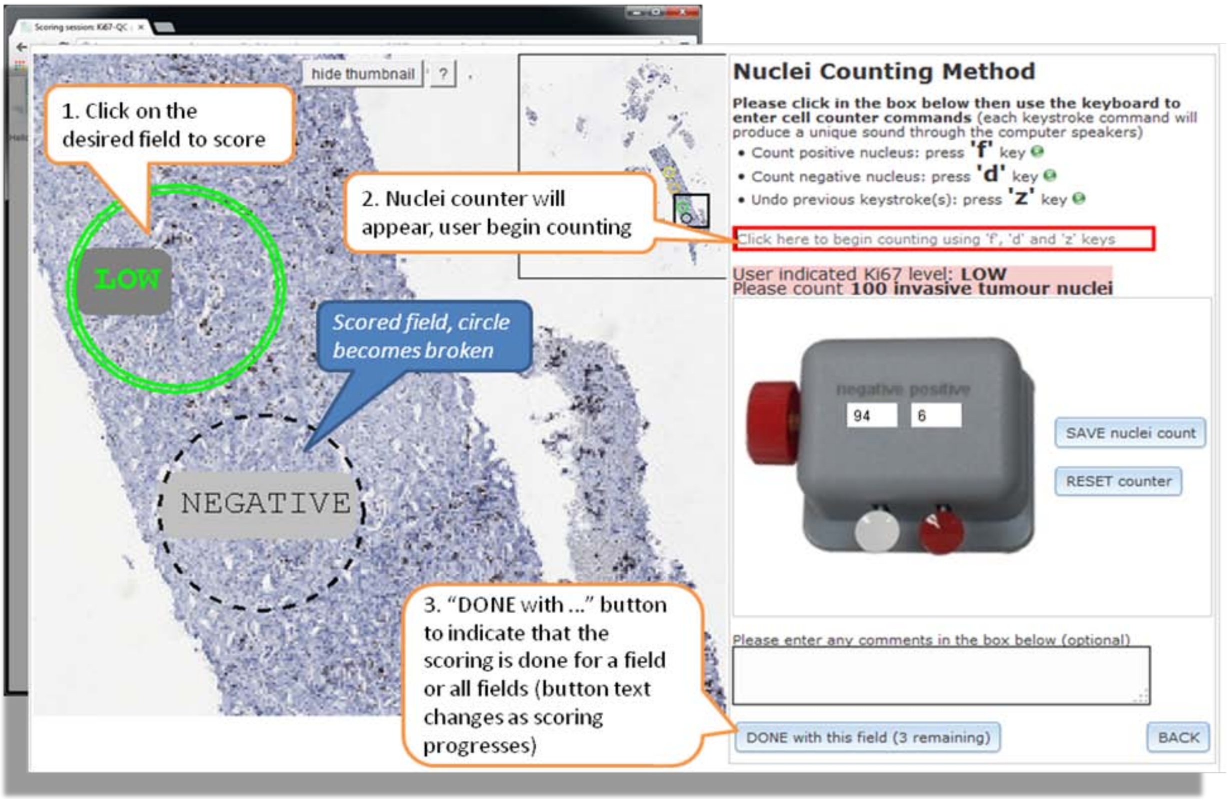

Circle formats:

- A **solid circle** indicates the field has not been scored yet.
- A **double-circle** indicates that you have selected / are scoring that field.
- A **broken (dashed) circle** indicates that the field has been scored.
- Each circle is also labeled with the Ki67 level it represents.

<u>Saving your work</u>:

Make sure to click "SAVE nuclei count" periodically. If you do not save after three hours, you will lose your work. A message will warn you 1 minute before the time-out.

Saving the nuclei count of a field may take <u>up to a minute</u>.

If, as you score, you wish to re-do your scoring of the field, click "RESET counter".

If you have any comments, please put them in the comment field.

When you reach 100 nuclei, the software will make a dinging sound, and a message will appear:

> *"100 nuclei have been counted. Please click on the 'done with this field' button to indicate that you have finished scoring this field."*

m) If you are done scoring the field, click **"DONE with this field"**.

n) Repeat the steps above until all circles have been scored. They will all appear as broken circles when scored.

o) Click "**done with all fields**". You have now scored the representative fields for this case.

10. Submitting your scores

a) Once you have finished scoring all 30 cases, a "Scoring session report" will appear **(Figure 11)**.

If you are ready to submit your scores, click "**submit scores**".

If you wish to review or revise the score for a core, you can click on that core's description in the "Description" column to return to the core.

Use the scroll arrows on the side of the table to scroll through the rows.

If you see only a partial row, click **CTRL** and **–** until you see rows.

b) To sign out, click on the **"Genetic Pathology Evaluation Centre" logo** at the top left of the window, and choose **"Sign out"**.

Figure 11. Reviewing, and then submitting, your scores

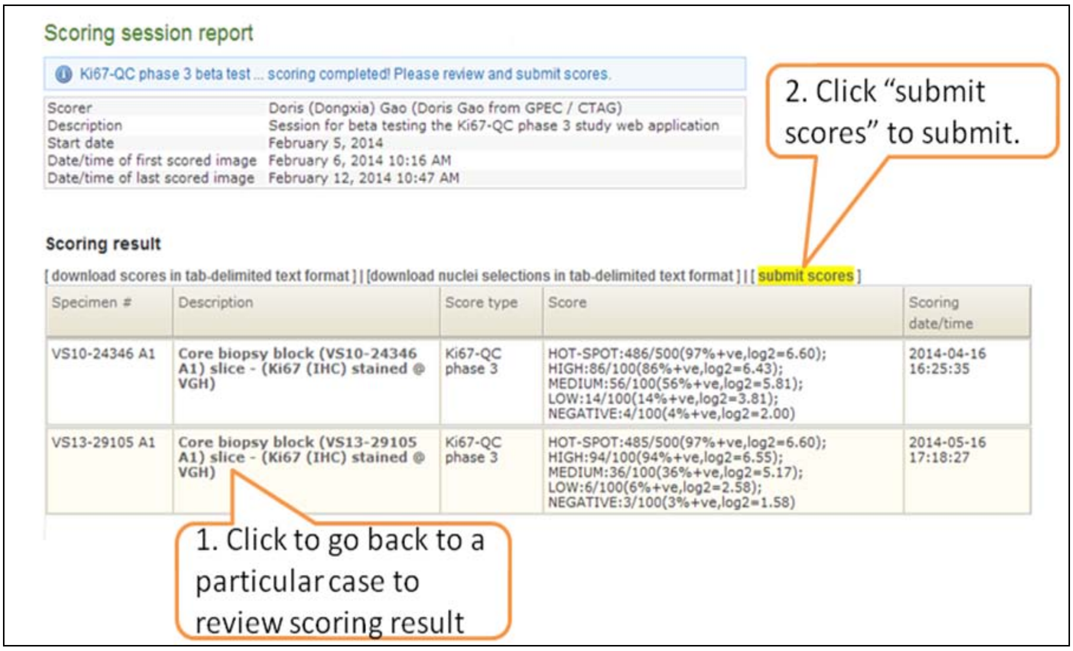

Appendix 1

What counts as "positive" Ki67 staining?

What counts as “positive”?

“Positive” = **<u>Any</u>** definite brown staining in the nucleus of an invasive breast cancer cell, above the surrounding background in cytoplasm and extracellular matrix.

- Cells with any degree/intensity of brown nuclear staining are considered positive.
- Cells showing only blue haematoxylin counterstain (i.e. an absence of brown nuclear staining) are considered negative.

Do not score DCIS. Only invasive carcinoma should be scored.

<u>Example images of positive and negative follow</u>.

Examples of range of staining levels that should be considered POSITIVE (red squares) for Ki67 (unmarked & marked side by side)

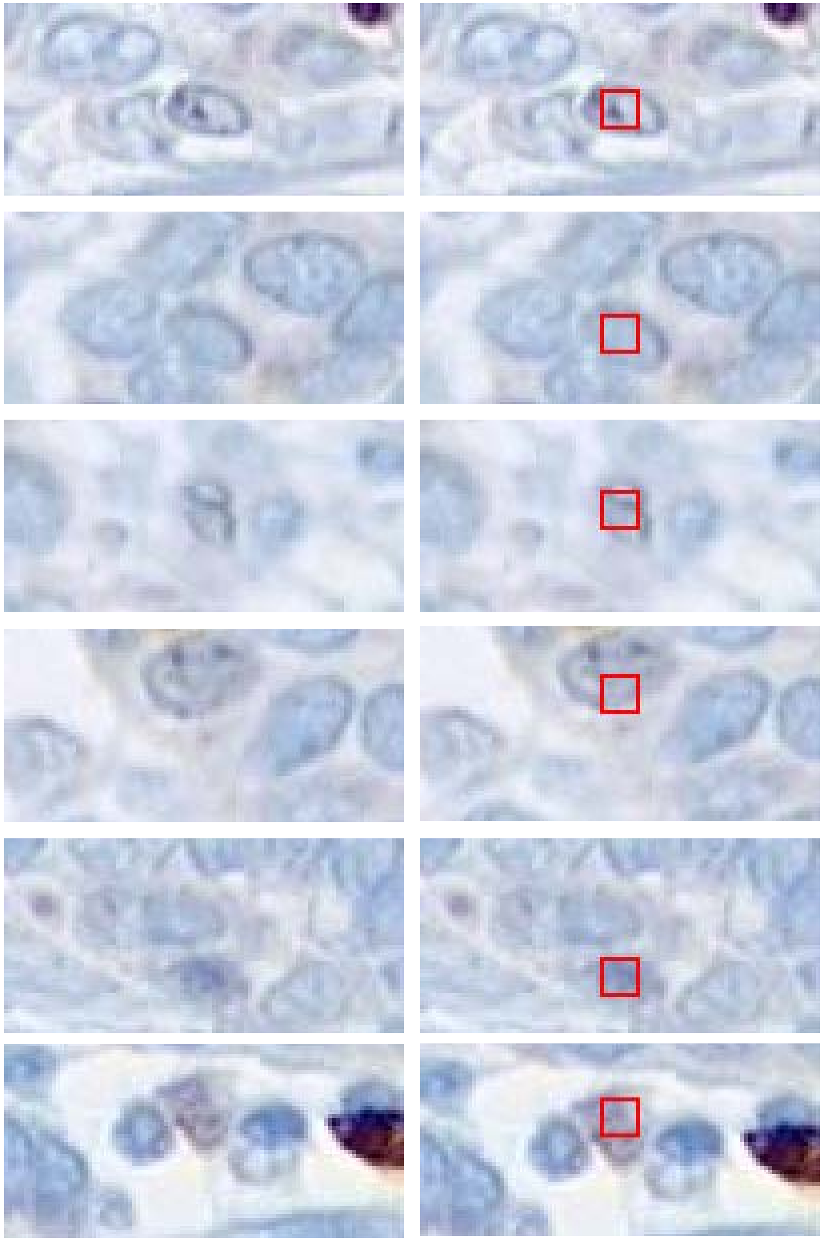

Positive nuclei are ordered from weakest to strongest brown stain.

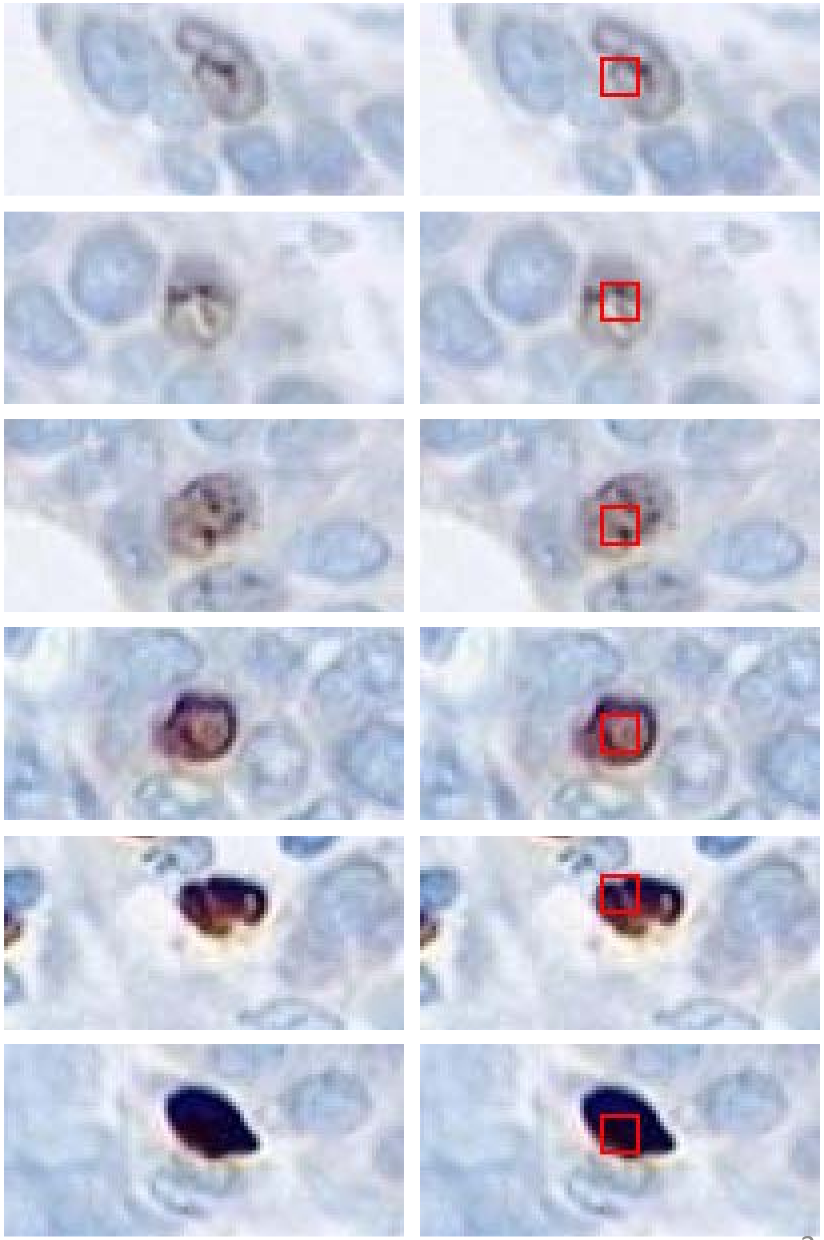

Examples of range of staining levels that should be considered NEGATIVE (green circles) for Ki67 (unmarked & marked side by side)

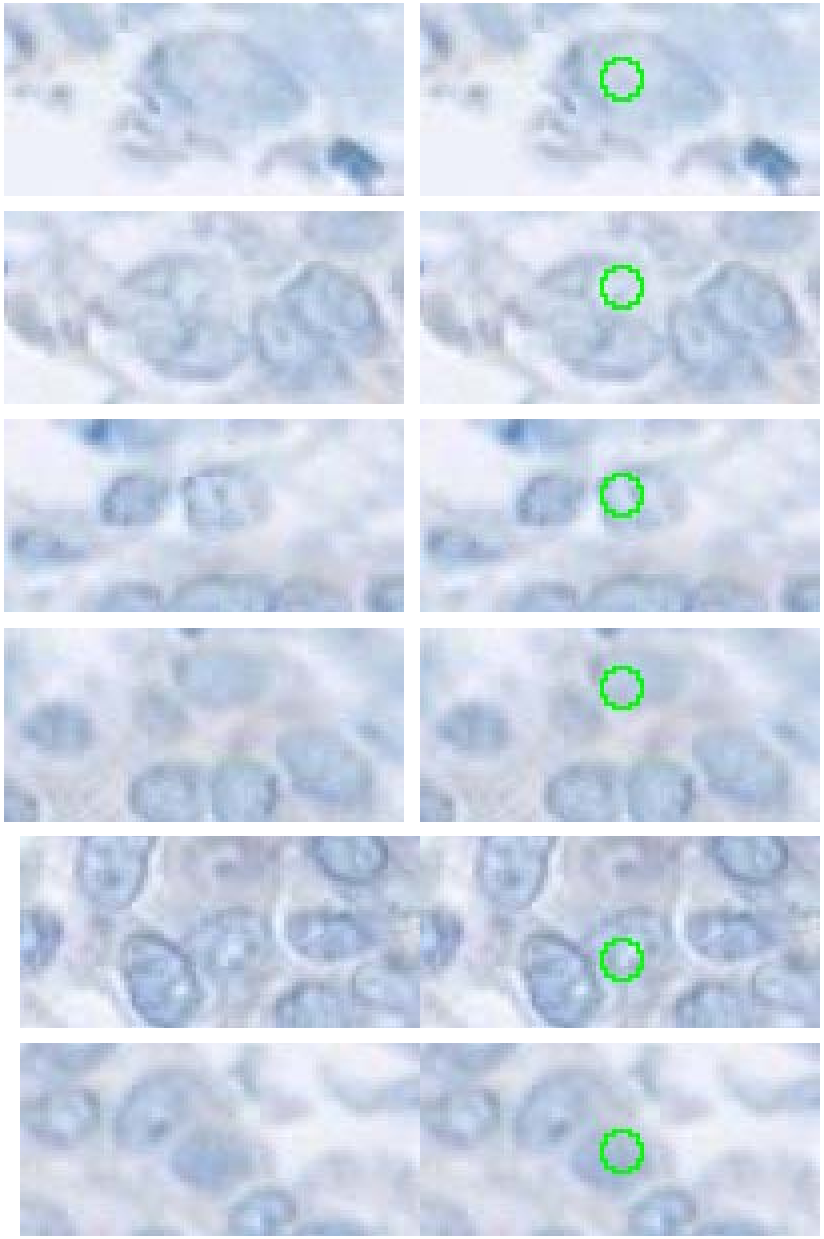

Negative nuclei are ordered from weakest to strongest blue stain.

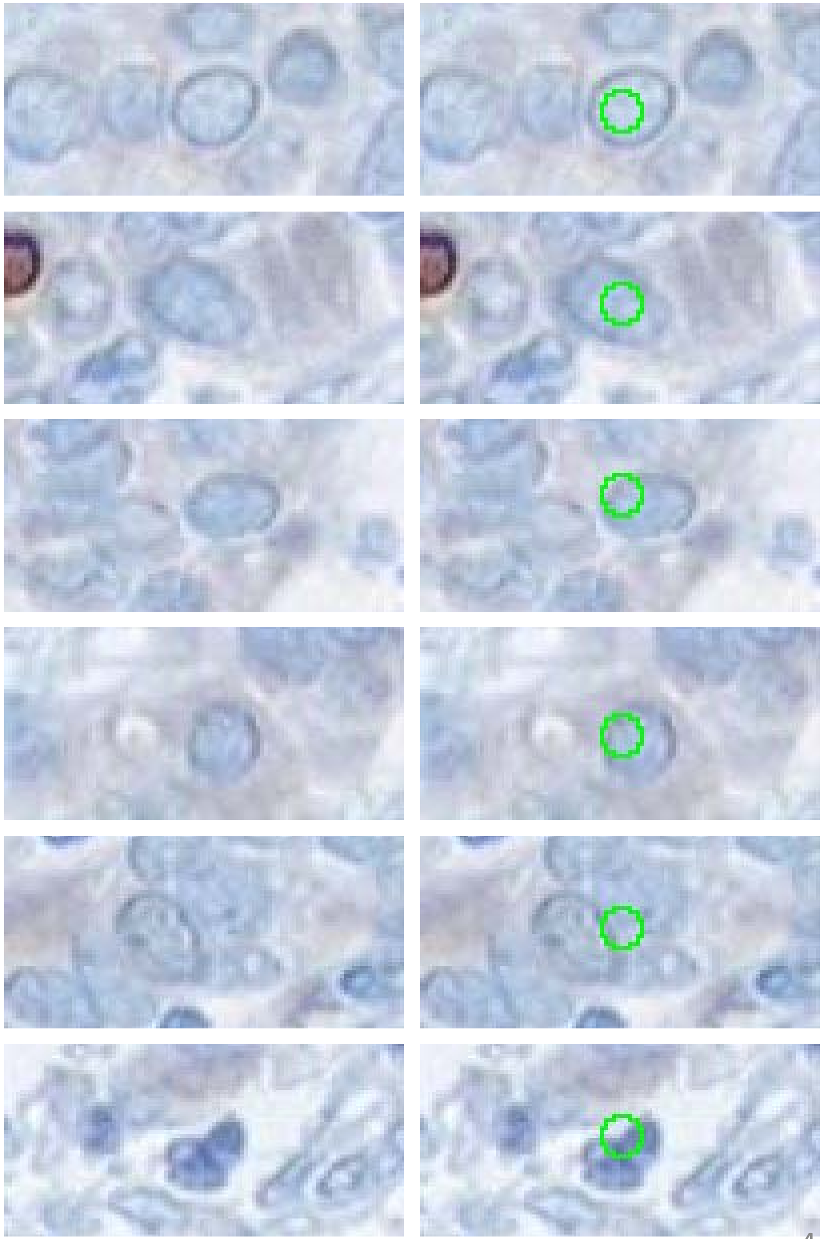

APPENDIX 2. Computer algorithm for assigning number of representative fields

For your information, below is a description of how the computer will select the number of fields per Ki67 level for the "selection of representative fields" step. This is a programmed algorithm.

Based on the percentages of Ki67 representation you indicated, the computer will assign up to 4 fields, as follows:

a) 1 field is assigned to each Ki67 level that you noted as being represented in the slide.

b) Then, the remaining fields are assigned as follows (note: just to those Ki67 levels you noted as being represented in the slide):

- **SCENARIO #1:** If you noted there were 3 different Ki67 levels represented in the slide, 1 field is now left of the possible 4 fields.

  → If there are **ties** among the percentages of Ki67 level representation (for example, 40% High, 40% Medium, 20% Low), the computer will not assign another field.

  → If there are **no ties** among the percentages of Ki67 level representation (for example, 10% High, 65% Medium, 25% Negative), the computer will assign the field to the Ki67 level with the highest percentage representation.

- **SCENARIO #2:** If you noted there were 2 different Ki67 levels represented in the slide, 2 fields are now left of the possible 4 fields.

  → If the difference in the % representation of the two Ki67 levels is less than 25%, the computer will assign 1 field to each level.

  → If the difference in the % representation of the two Ki67 levels is greater than or equal to 25%, the computer will assign the 2 fields to the highest % level.

- **SCENARIO #3:** If you noted there was only 1 Ki67 level represented in the slide (i.e., a homogenous sample with a certain Ki67 at 100% representation), then 3 fields will be left.

  → The computer will assign all 3 fields to that one Ki67 level.
