# Supplementary material for: Analytical validation of a standardized scoring protocol for Ki67: phase 3 of an international multicenter collaboration
Source: NPJ Breast Cancer. 2016 May 18;2:16014–. doi: 10.1038/npjbcancer.2016.14 (PMC5515324; doi:10.1038/npjbcancer.2016.14)
Supplement: Supplementary Figures [file npjbcancer201614-s4.pdf]

Analytical validation of a standardized scoring protocol for Ki67: phase 3 of an international multicenter collaboration.

Supplemental document: exploratory examination of scoring fields

- **Hypothesis**: detail examination of scoring fields may reveal potential sources of variability that could help explain the observed discrepancies in Ki67 scores
- **Method**: select and examine scoring fields of cases where labs gave fairly discrepant scores
- **Results**: five sources of variability were identified
    1. counting of ductal carcinoma in situ (DCIS)
    2. counting of stromal cells
    3. positive nuclei localized within a part of the scoring field
    4. labs measuring higher or lower than others tended to do so relatively consistently – labs may need recalibration?
    5. hot-spot score discrepancy seems to be driven (partly) by field selection

1. Counting of DCIS

- TB250 (hot-spot scores)
- Lab T gave significantly higher Ki67 score (42%) compared to other labs (*group 3 median: 19%*)
- It appears that DCIS may have been scored. Examination of the corresponding p63 image supported this view (*please refer to the next slide*).

(median score)	GROUP 1										GROUP 2		GROUP 3										
TB196 (7)	4	9	8	7	6	7	11	11	4	15	5	16	3	10	7	17	5	14	3	4	6	28	15:6:1
TB040 (8)	7	7	8	12	7	9	5	8	2	15	13	9	6	10	10	6	8	5	11	5	11	14	14:8:0
TB374 (8)	2	8	7	7	9	7	7	12	6	19	11	12	4	7	8	9	10	10	10	3	6	18	15:7:0
TB083 (10)	6	4	10	11	12	6	12	16	5	11	13	9	10	8	11	7	6	10	11	6	10	31	10:11:1
TB107 (11)	7	5	13	10	8	9	6	12	9	15	17	15	10	12	11	11	11	16	12	8	14	28	7:14:1
TB016 (12)	9	8	13	14	10	12	11	12	11	13	15	14	6	12	13	18	6	9	11	10	11	30	5:16:1
TB113 (12)	9	13	15	18	9	11	19	11	18	26	16	11	12	7	6	7	8	13	13	8	15	27	7:13:2
TB634 (15)	9	16	18	20	19	20	14	20	22	30	14	17	13	13	11	15	12	13	14	15	15	28	1:17:4
TB193 (18)	10	14	18	21	24	18	15	19	22	32	20	23	11	16	15	16	14	19	15	19	21	33	0:14:8
TB036 (19)	15	14	19	22	21	17	19	20	21	29	21	23	14	13	14	15	9	15	20	15	23	24	1:13:8
TB112 (20)	21	19	15	29	14	28	21	18	33	38	15	18	25	20	20	18	13	16	17	14	28	[illegible]	0:11:11
TB250 (21)	6	21	26	24	26	22	21	23	26	33	21	26	8	19	21	20	25	19	19	16	18	42	[illegible]:6:14
TB286 (26)	14	20	23	29	29	26	25	26	31	38	22	31	23	13	28	25	29	26	25	84	23	[illegible]	0:2:20
TB192 (27)	18	15	23	28	28	40	21	41	27	39	38	32	19	25	22	23	25	28	28	18	30	44	0:4:18
TB082 (29)	15	25	28	27	27	20	35	32	27	31	32	36	22	27	23	29	21	35	32	31	41	51	0:2:20
TB033 (31)	19	18	21	30	30	29	34	32	34	37	34	39	31	32	30	31	33	31	26	30	40	42	0:2:20
TB090 (33)	23	28	31	36	37	34	33	38	29	50	41	40	31	24	22	32	26	25	34	27	37	49	0:0:22
TB468 (34)	21	28	32	35	37	46	31	30	53	51	38	43	33	43	43	26	30	31	33	33	37	43	0:0:22
TB022 (34)	22	30	36	32	34	31	32	36	35	37	43	40	23	30	35	24	38	31	34	30	49	51	0:0:22
TB105 (37)	31	33	40	48	41	37	38	44	43	47	38	39	28	37	27	36	39	34	32	35	32	46	0:0:22
TB067 (41)	30	27	43	42	45	37	40	44	37	46	41	44	45	23	36	29	38	38	36	45	56	42	0:0:22
TB203 (42)	27	42	44	52	52	58	47	49	51	54	39	52	39	33	30	28	40	35	38	41	43	44	0:0:22
TB310 (45)	29	44	47	49	56	44	44	50	55	54	44	50	43	45	37	37	44	44	41	85	51	61	0:0:22
TB405 (47)	36	36	47	37	32	51	30	50	53	55	47	45	32	41	44	47	46	51	35	82	54	59	0:0:22
TB381 (50)	20	50	36	66	63	49	61	50	46	65	51	49	45	54	51	41	33	46	47	85	49	59	0:1:21
TB319 (55)	47	55	54	68	65	60	58	59	55	66	47	54	53	52	49	52	57	56	50	81	70	47	0:0:22
TB077 (61)	47	50	60	58	72	60	67	57	63	70	43	55	65	58	63	61	71	57	67	62	74	69	0:0:22
TB633 (62)	62	54	65	70	65	61	66	71	70	72	58	61	48	58	61	56	57	53	61	84	64	73	0:0:22
TB245 (76)	73	71	85	71	89	82	86	75	87	73	65	77	76	70	76	69	86	66	85	83	90	75	0:0:22
TB460 (93)	95	94	93	98	99	98	99	90	98	91	84	93	88	87	91	91	94	93	91	91	95	86	0:0:22
Specimen number	A	I	C	E	G	B	F	J	D	H	L	K	R	O	P	V	U	Q	S	N	M	T	

Laboratory

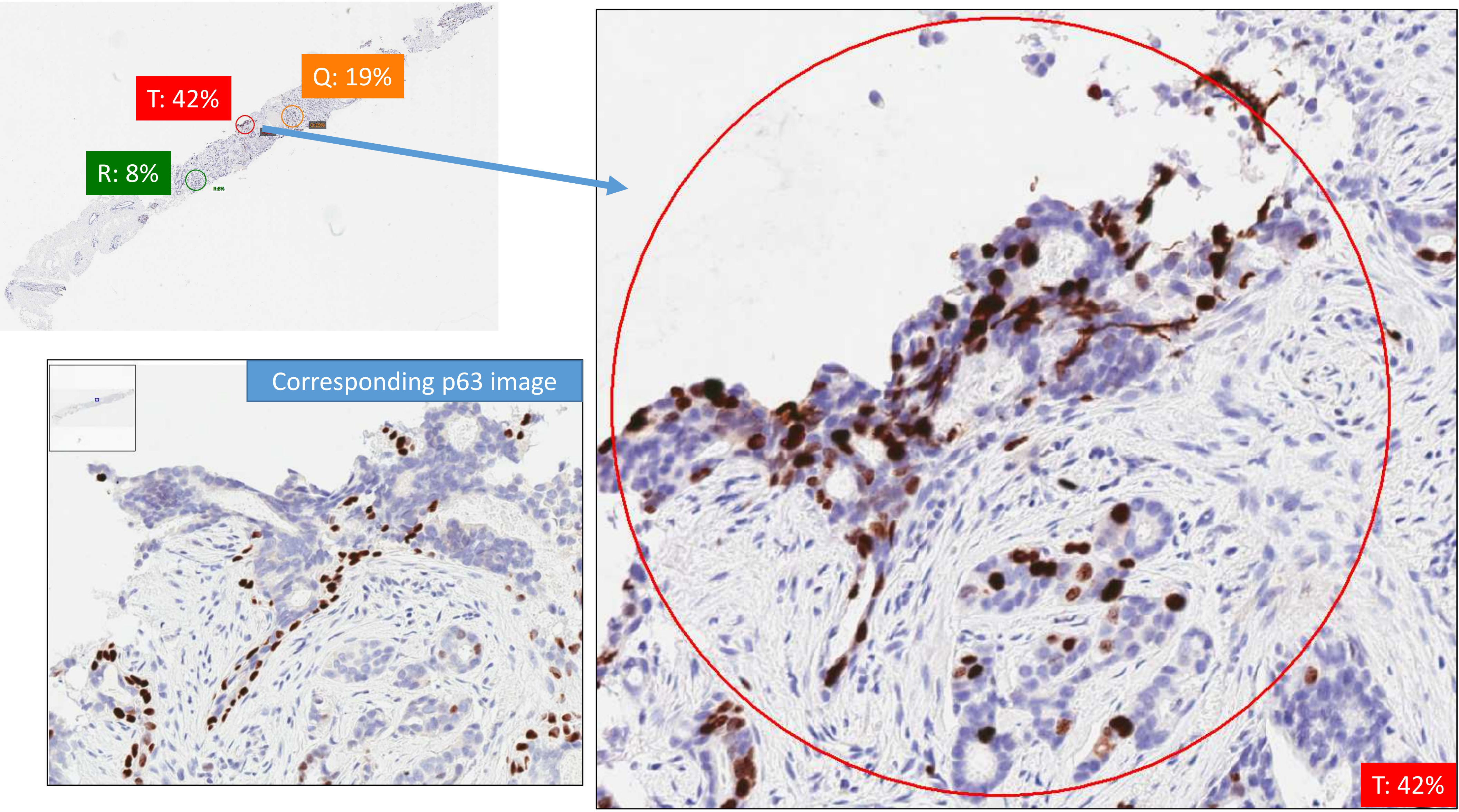
Q: 19%
T: 42%
R: 8%
Corresponding p63 image
T: 42%

2. Counting of stromal cells

- TB196 (hot-spot scores)
- Lab T gave significantly higher Ki67 score (28%) compared to other labs (*group 3 median: 7%*)
- It seems that some of the stromal cells have been scored, contributing to the higher count (*please refer to the next slide*).

Specimen number (median score)	GROUP 1										GROUP 2		GROUP 3										
Laboratory	A	I	C	E	G	B	F	J	D	H	L	K	R	O	P	V	U	Q	S	N	M	T	
TB196 (7)	4	9	8	7	6	7	11	11	4	15	5	16	3	10	7	17	5	14	3	4	6	28	[illegible]5:6:1
TB040 (8)	7	7	8	12	7	9	5	8	2	15	13	9	6	10	10	6	8	5	11	5	11	[illegible]	14:8:0
TB374 (8)	2	8	7	7	9	7	7	12	6	19	11	12	4	7	8	9	10	10	10	3	6	18	15:7:0
TB083 (10)	6	4	10	11	12	6	12	16	5	11	13	9	10	8	11	7	6	10	11	6	10	31	10:11:1
TB107 (11)	7	5	13	10	8	9	6	12	9	15	17	15	10	12	11	11	11	16	12	8	14	28	7:14:1
TB016 (12)	9	8	13	14	10	12	11	12	11	13	15	14	6	12	13	18	6	9	11	10	11	30	5:16:1
TB113 (12)	9	13	15	18	9	11	19	11	18	26	16	11	12	7	6	7	8	13	13	8	15	27	7:13:2
TB634 (15)	9	16	18	20	19	20	14	20	22	30	14	17	13	13	11	15	12	13	14	15	15	28	1:17:4
TB193 (18)	10	14	18	21	24	18	15	19	22	32	20	23	11	16	15	16	14	19	15	19	21	33	0:14:8
TB036 (19)	15	14	19	22	21	17	19	20	21	29	21	23	14	13	14	15	9	15	20	15	23	24	1:13:8
TB112 (20)	21	19	15	29	14	28	21	18	33	38	15	18	25	20	20	18	13	16	17	14	28	27	0:11:11
TB250 (21)	6	21	26	24	26	22	21	23	26	33	21	26	8	19	21	20	25	19	19	16	18	42	2:6:14
TB286 (26)	14	20	23	29	29	26	25	26	31	38	22	31	23	13	28	25	29	26	25	84	23	38	0:2:20
TB192 (27)	18	15	23	28	28	40	21	41	27	39	38	32	19	25	22	23	25	28	28	18	30	44	0:4:18
TB082 (29)	15	25	28	27	27	20	35	32	27	31	32	36	22	27	23	29	21	35	32	31	41	51	0:2:20
TB033 (31)	19	18	21	30	30	29	34	32	34	37	34	39	31	32	30	31	33	31	26	30	40	42	0:2:20
TB090 (33)	23	28	31	36	37	34	33	38	29	50	41	40	31	24	22	32	26	25	34	27	37	49	0:0:22
TB468 (34)	21	28	32	35	37	46	31	30	53	51	38	43	33	43	43	26	30	31	33	33	37	43	0:0:22
TB022 (34)	22	30	36	32	34	31	32	36	35	37	43	40	23	30	35	24	38	31	34	30	49	51	0:0:22
TB105 (37)	31	33	40	48	41	37	38	44	43	47	38	39	28	37	27	36	39	34	32	35	32	46	0:0:22
TB067 (41)	30	27	43	42	45	37	40	44	37	46	41	44	45	23	36	29	38	38	36	45	56	42	0:0:22
TB203 (42)	27	42	44	52	52	58	47	49	51	54	39	52	39	33	30	28	40	35	38	41	43	44	0:0:22
TB310 (45)	29	44	47	49	56	44	44	50	55	54	44	50	43	45	37	37	44	44	41	85	51	61	0:0:22
TB405 (47)	36	36	47	37	32	51	30	50	53	55	47	45	32	41	44	47	46	51	35	82	54	59	0:0:22
TB381 (50)	20	50	36	66	63	49	61	50	46	65	51	49	45	54	51	41	33	46	47	85	49	59	0:1:21
TB319 (55)	47	55	54	68	65	60	58	59	55	66	47	54	53	52	49	52	57	56	50	81	70	47	0:0:22
TB077 (61)	47	50	60	58	72	60	67	57	63	70	43	55	65	58	63	61	71	57	67	62	74	69	0:0:22
TB633 (62)	62	54	65	70	65	61	66	71	70	72	58	61	48	58	61	56	57	53	61	84	64	73	0:0:22
TB245 (76)	73	71	85	71	89	82	86	75	87	73	65	77	76	70	76	69	86	66	85	83	90	75	0:0:22
TB460 (93)	95	94	93	98	99	98	99	90	98	91	84	93	88	87	91	91	94	93	91	91	95	86	0:0:22

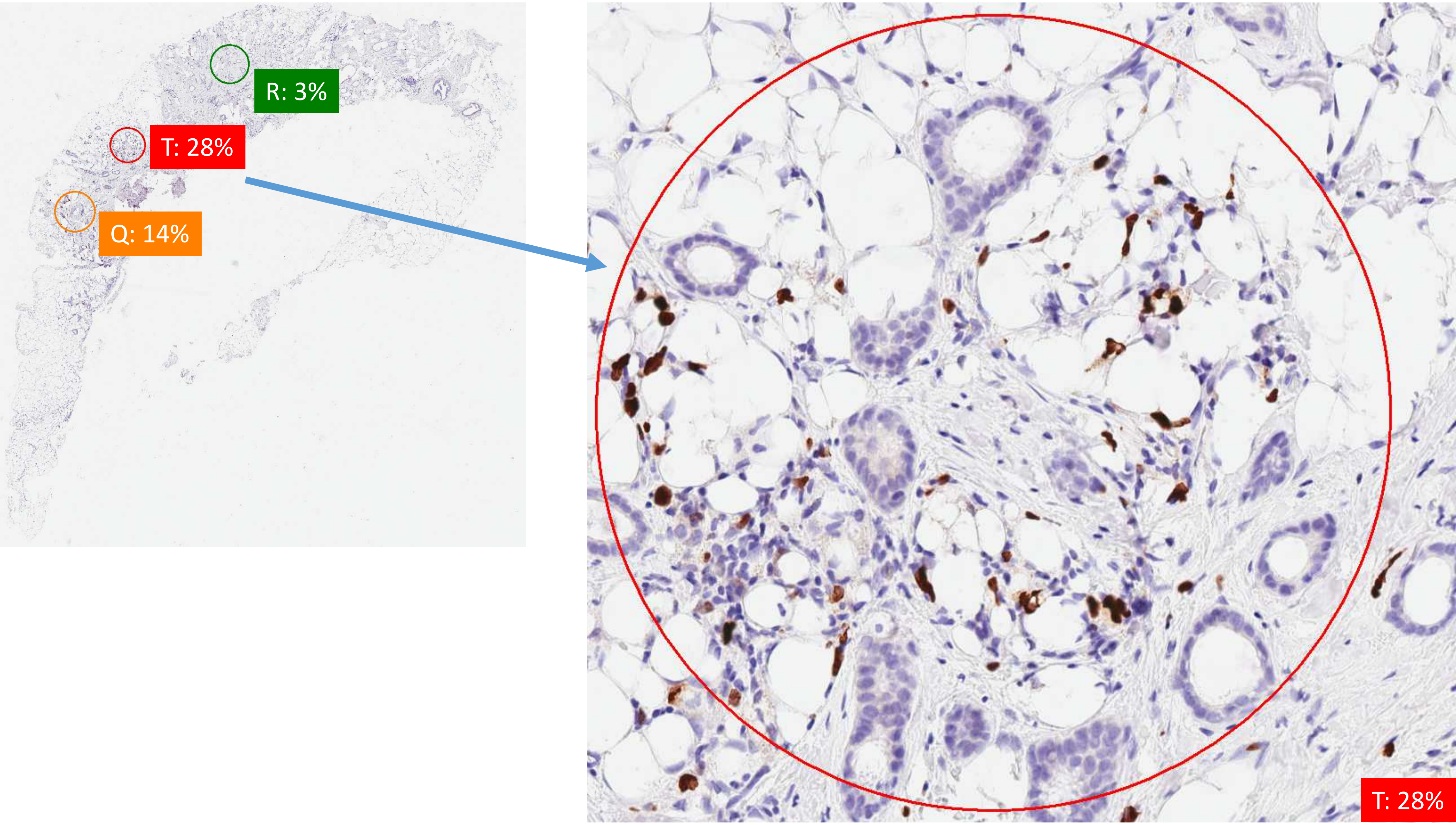
R: 3%
T: 28%
Q: 14%
T: 28%

3. Positive nuclei localized within a part of the scoring field

- TB193 – group 1 slide
- All labs selected a similar hot-spot field.
- However, score among group 1 ranges from 10% to 32% (mean: 19%)
- One possible cause: positive nuclei localized within a part of the scoring field (*please refer to the next slide*).

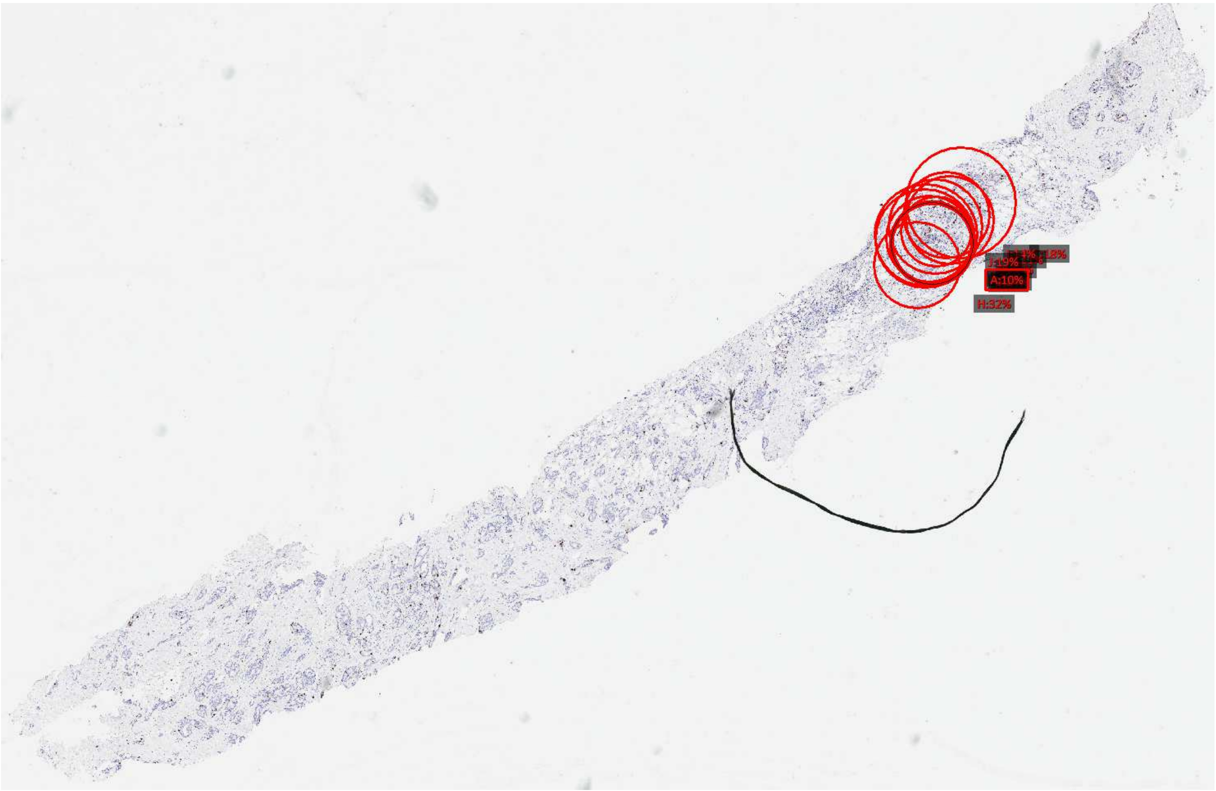

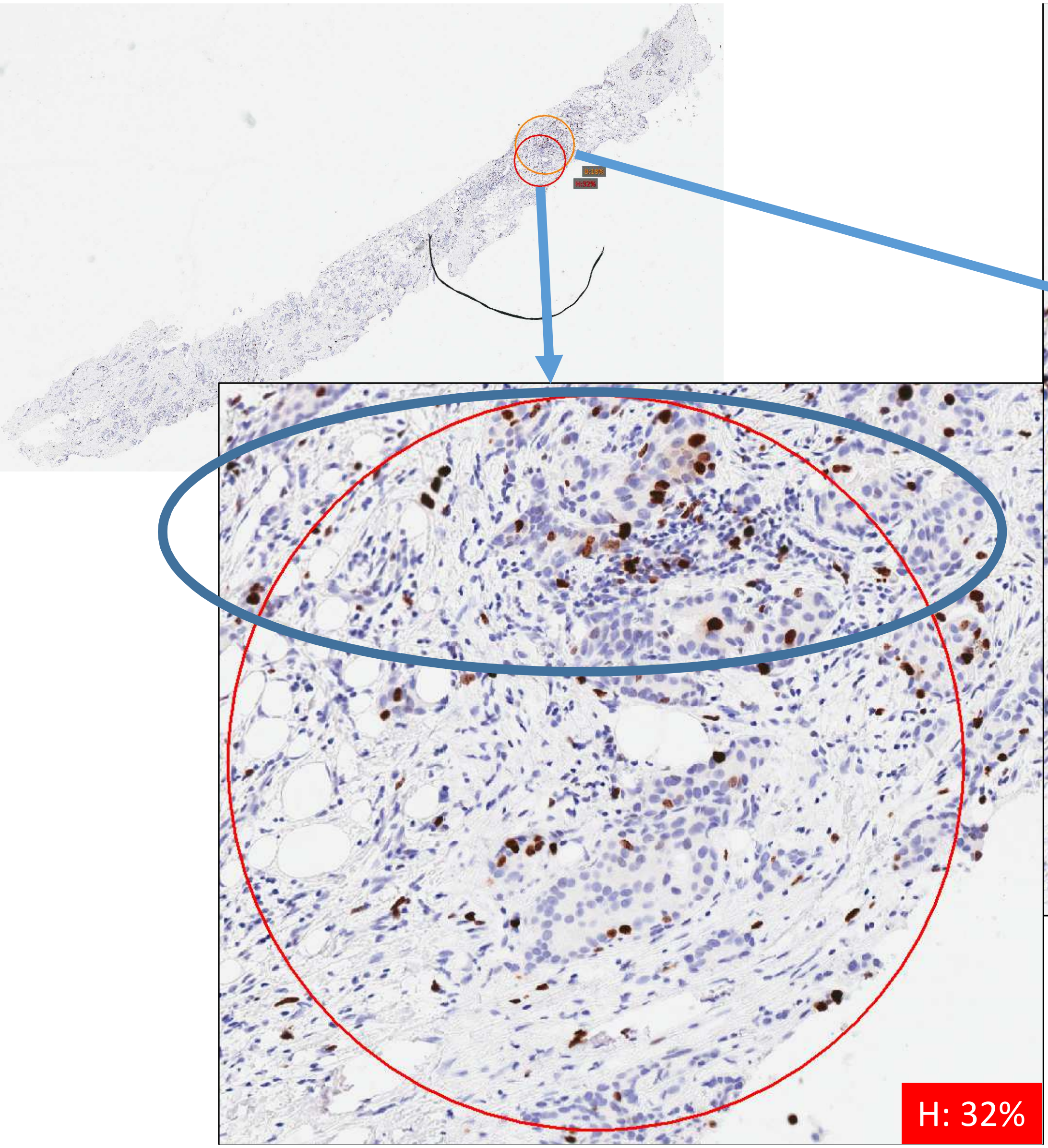

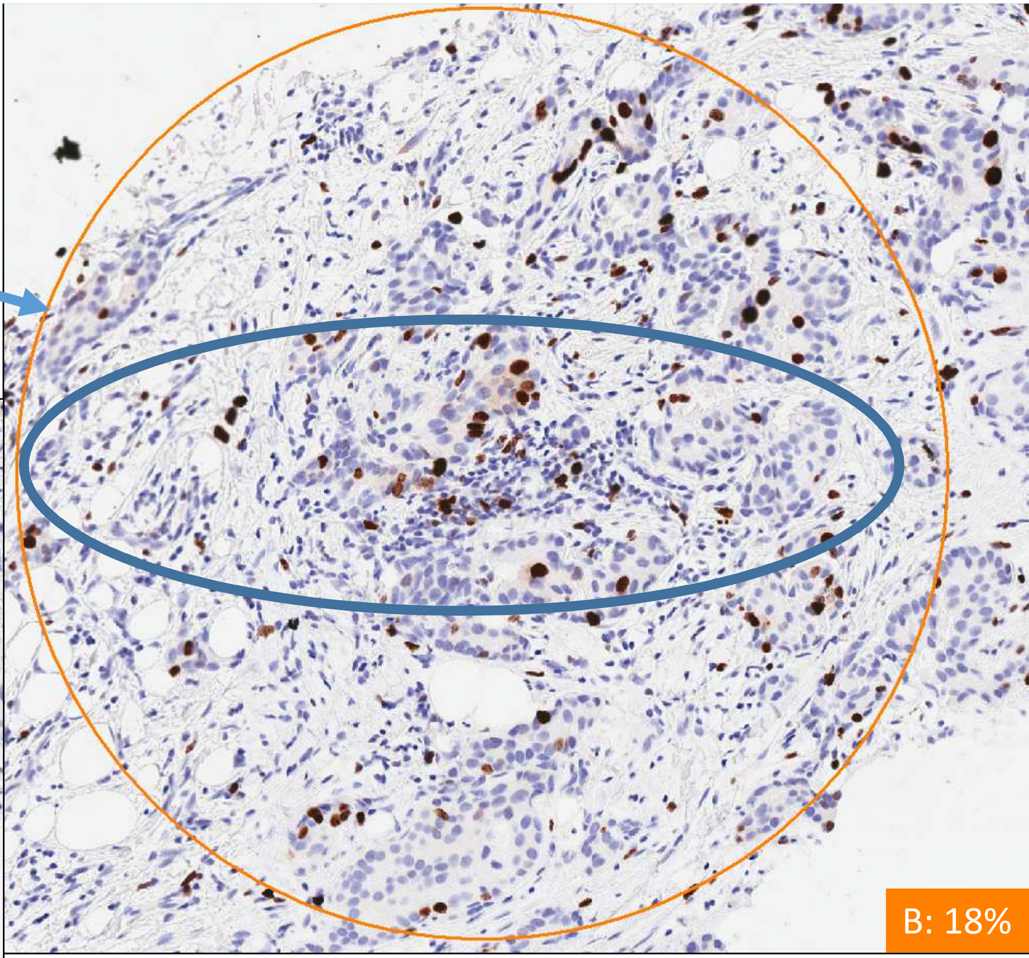

Positive nuclei are more concentrated on the top of the scoring field chosen by lab H compared to the field selected by lab B.

4. Labs measuring higher or lower than others tended to do so relatively consistently – labs may need recalibration?

- The following are three cases scored by lab R and T using the global method. They show that the scores given by lab R are fairly consistently lower compared to lab T's scores when they scored the same (or approximately the same) fields.

Specimen number (median score)	A	I	F	B	G	D	J	C	E	H	L	K	R	P	O	V	S	U	Q	N	T	M	
	GROUP 1										GROUP 2		GROUP 3										
TB374 (4)	2	2	4	6	6	4	2	3	4	6	4	4	4	4	5	1	2	2	5	4	4	4	22:0:0
TB196 (5)	4	4	2	5	6	3	3	4	6	6	4	8	3	6	8	5	1	2	9	6	17	4	21:1:0
TB040 (5)	3	3	2	5	5	4	2	6	9	15	12	7	4	8	5	3	6	9	5	4	10	9	20:2:0
TB083 (7)	4	5	5	5	10	6	8	7	7	11	11	10	4	7	8	6	8	7	5	5	17	12	18:4:0
TB113 (8)	7	10	6	6	7	13	5	5	9	18	10	7	7	8	5	6	10	6	9	8	14	9	18:4:0
TB107 (8)	4	6	6	7	8	6	10	6	6	12	13	8	7	7	14	11	13	4	14	8	18	12	14:8:0
TB112 (9)	7	12	8	10	7	9	13	8	9	16	12	9	6	8	8	6	12	10	9	14	15	8	14:8:0
TB016 (10)	7	9	10	10	12	11	15	14	10	20	12	13	9	10	9	8	11	7	8	6	18	13	12:10:0
TB193 (11)	8	10	8	11	16	12	18	10	15	26	12	15	9	8	8	11	11	7	11	15	20	15	6:14:2
TB634 (13)	8	12	8	10	13	13	15	12	12	23	9	13	11	12	9	15	17	12	16	28	24	19	4:15:3
TB250 (14)	12	11	10	15	13	13	19	15	15	20	16	10	9	14	14	13	15	12	12	21	17	16	2:18:2
TB022 (19)	14	21	22	18	18	8	24	22	18	25	18	20	15	16	16	18	20	21	22	13	24	30	1:12:9
TB036 (19)	15	16	16	18	23	19	21	20	26	30	24	24	14	12	16	16	21	14	18	19	21	25	0:13:9
TB033 (20)	19	17	20	19	22	21	26	26	22	30	26	28	14	20	10	20	19	20	22	18	29	28	1:10:11
TB286 (21)	15	11	16	18	24	26	22	20	21	24	20	29	15	16	24	20	13	28	19	62	28	28	0:10:12
TB192 (21)	9	19	13	31	25	26	25	31	28	33	28	18	18	17	17	15	18	18	21	37	27	22	1:9:12
TB082 (26)	17	17	16	22	26	23	34	26	26	29	28	34	13	24	22	30	29	27	20	22	34	39	0:5:17
TB090 (28)	14	23	24	24	30	31	27	25	34	38	36	38	14	16	30	31	32	24	31	27	26	44	0:3:19
TB381 (29)	25	22	26	38	38	42	34	28	38	37	27	24	30	31	22	24	29	20	27	67	30	33	0:0:22
TB468 (32)	25	28	21	41	31	43	32	33	41	47	24	30	20	32	32	32	29	30	25	44	34	36	0:1:21
TB405 (33)	26	25	23	38	36	35	42	31	33	43	38	29	16	22	32	34	30	33	28	64	33	38	0:1:21
TB105 (34)	25	29	26	30	37	31	46	32	43	41	30	38	24	32	34	30	34	43	38	36	48	40	0:0:22
TB067 (34)	30	32	32	34	41	27	34	41	33	42	32	38	29	27	32	30	36	49	26	38	40	55	0:0:22
TB203 (36)	29	40	27	51	37	46	36	52	45	45	31	43	29	22	21	32	31	36	30	64	44	36	0:0:22
TB077 (39)	30	37	48	52	38	33	40	50	51	31	38	43	33	38	33	43	39	52	32	62	52	68	0:0:22
TB310 (41)	32	41	34	48	55	42	42	46	46	53	36	37	29	28	36	38	41	42	39	85	42	46	0:0:22
TB633 (49)	41	44	44	54	52	45	30	42	58	58	51	57	39	43	47	43	60	46	53	90	50	58	0:0:22
TB319 (51)	41	45	49	66	60	59	56	49	58	61	42	50	51	48	44	48	56	52	48	82	48	67	0:0:22
TB245 (70)	68	70	70	78	68	70	67	76	73	75	58	61	77	66	54	61	76	78	50	83	66	77	0:0:22
TB460 (88)	89	84	90	74	98	97	94	88	92	77	82	90	87	89	86	84	84	79	85	94	79	95	0:0:22

Laboratory

Example case 1 of 3: TB192

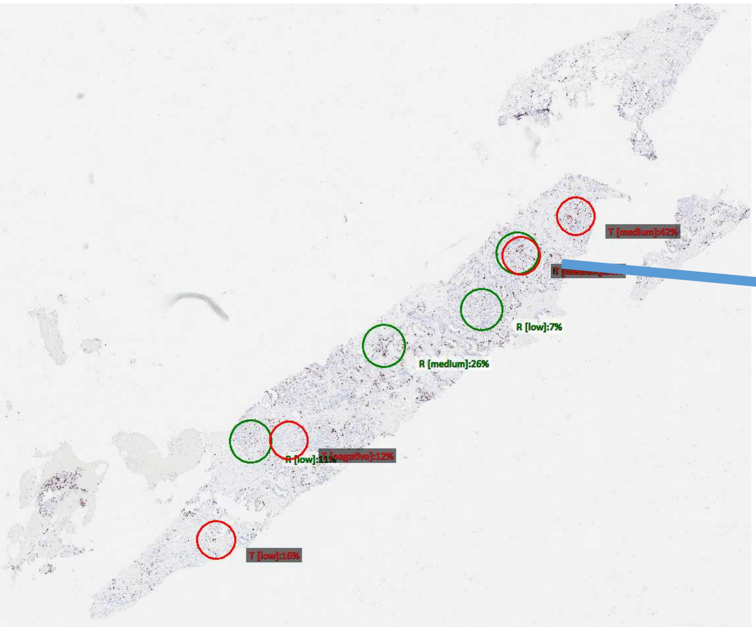

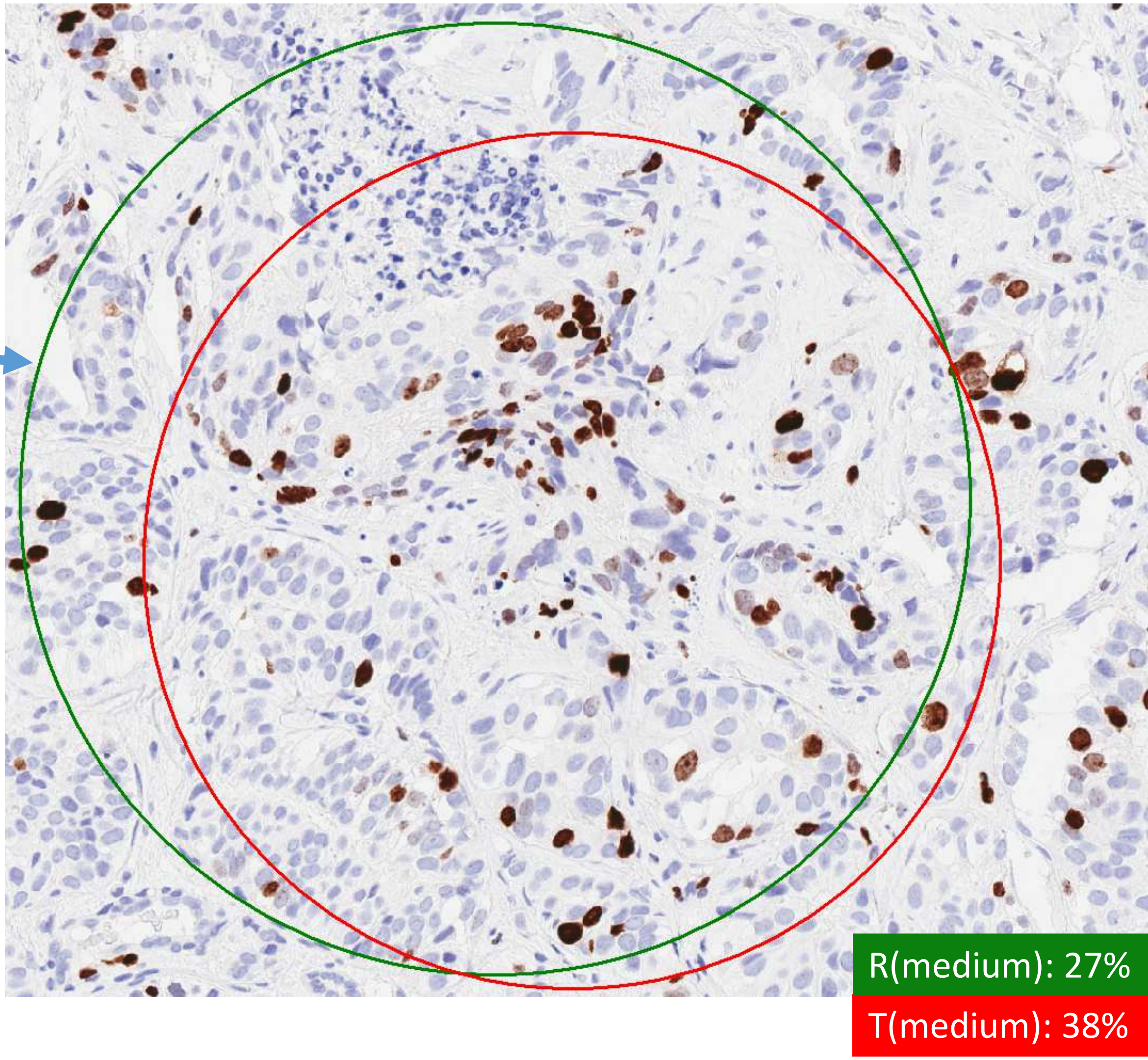

Example case 2 of 3: TB193

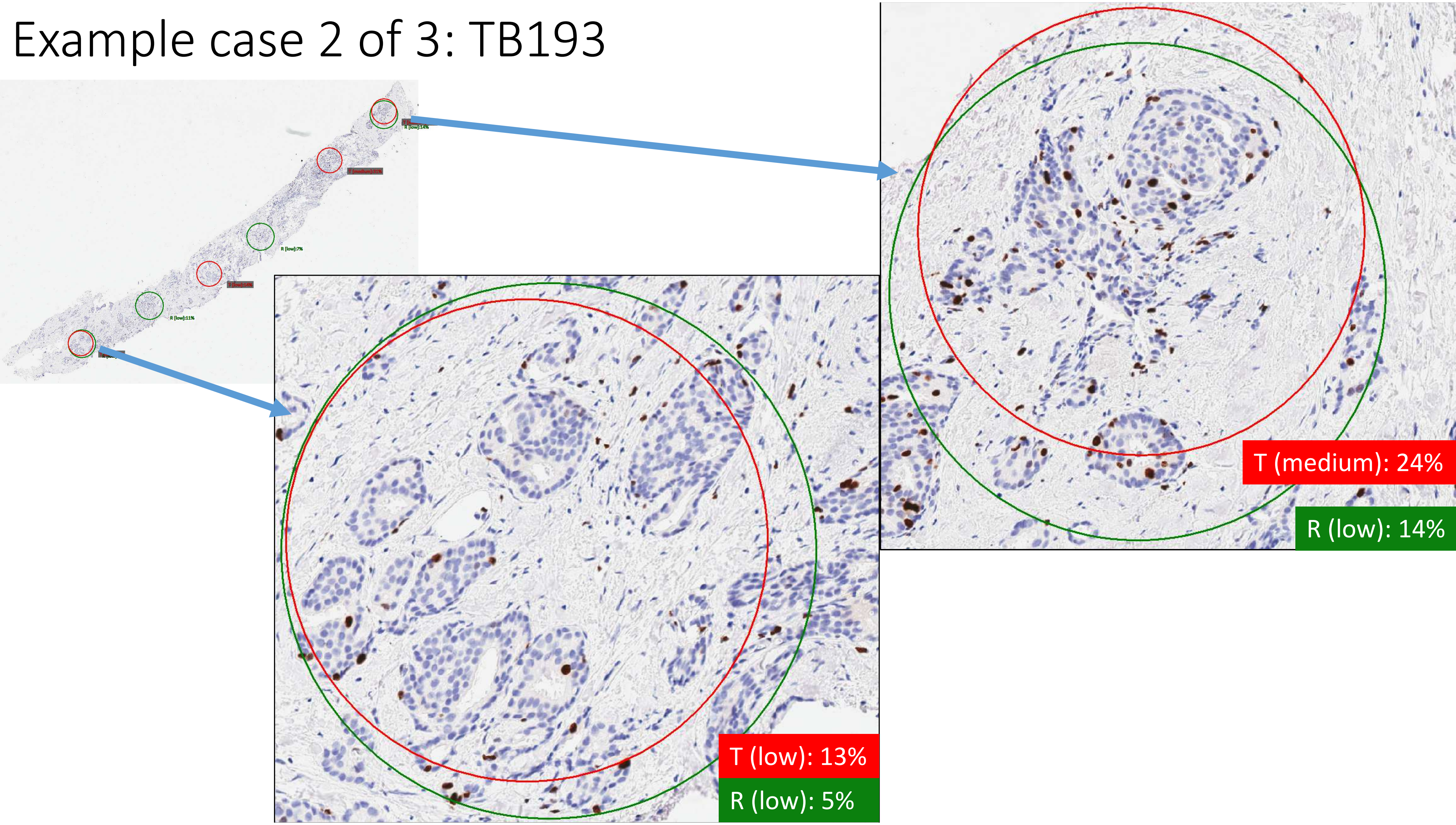

Example case 3 of 3: TB374

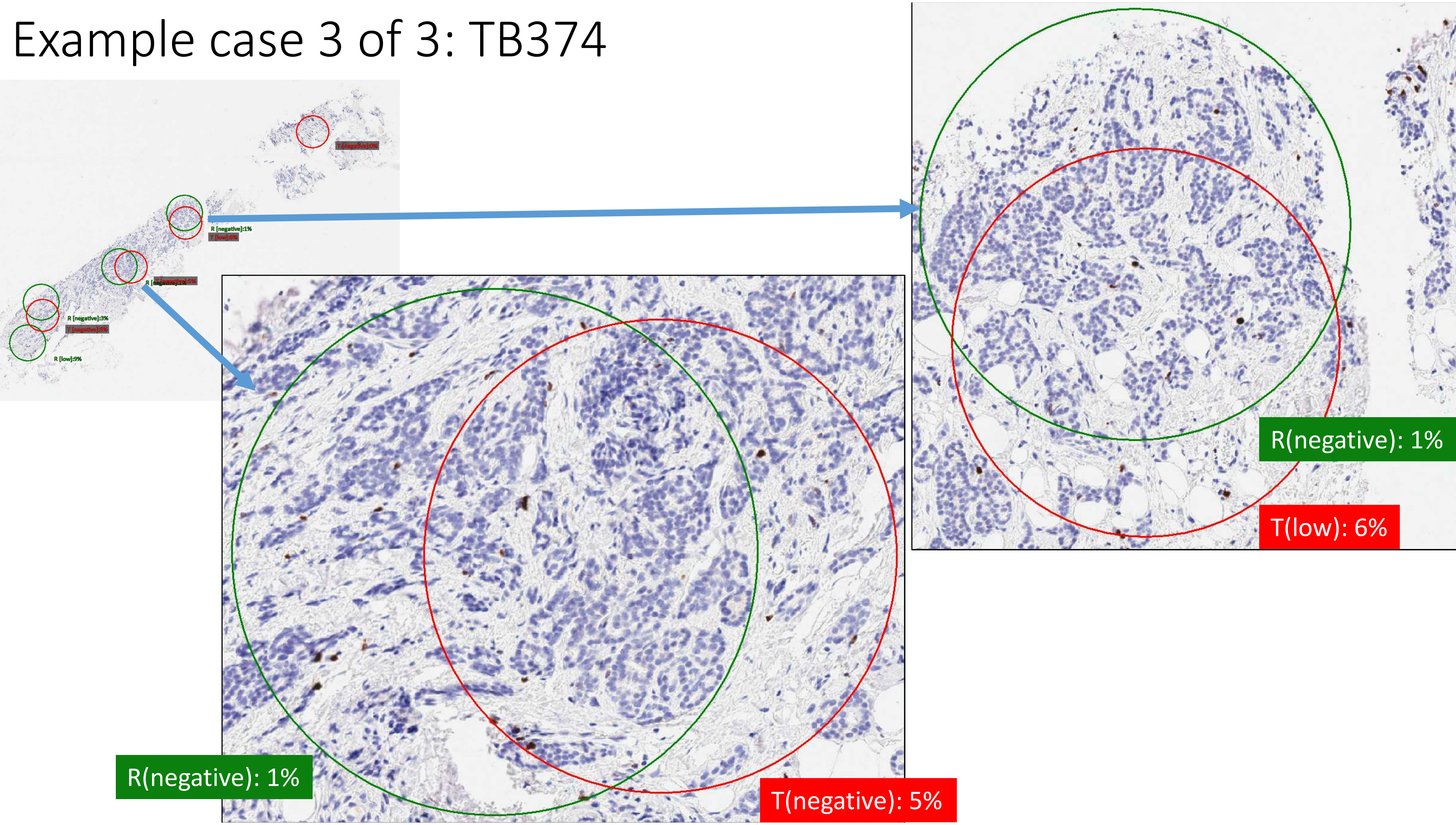

5. Hot-spot score discrepancy seems to be driven (partly) by field selection

- The following two cases are example of hot-spot scores being fairly consistent if similar hot-spot fields were selected.

Example 1 of 2: consistent hot-spot scores on similar hot-spot fields.
TB016 (group 3)

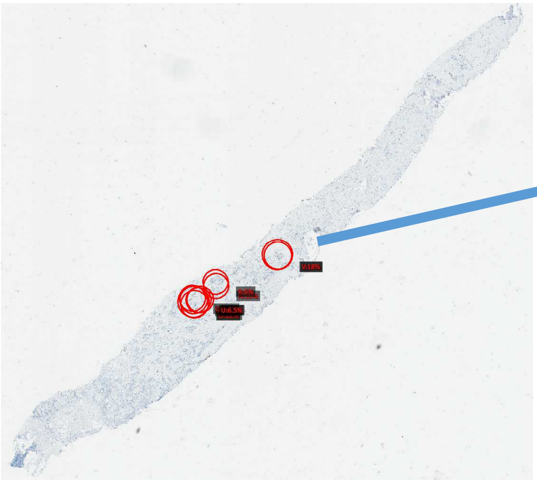

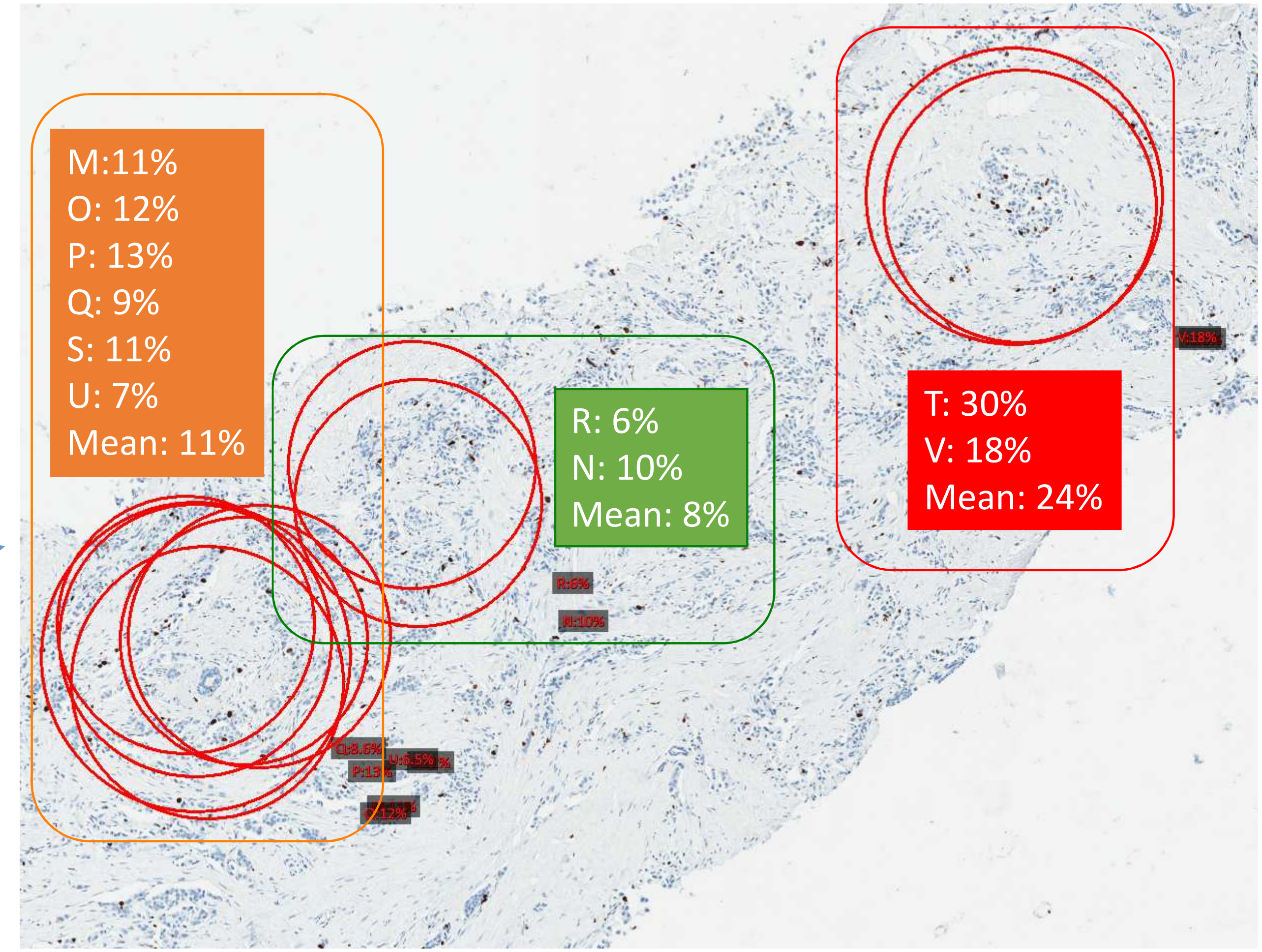

Example 2of 2: consistent hot-spot scores on similar hot-spot fields
TB250 (group 1&3)

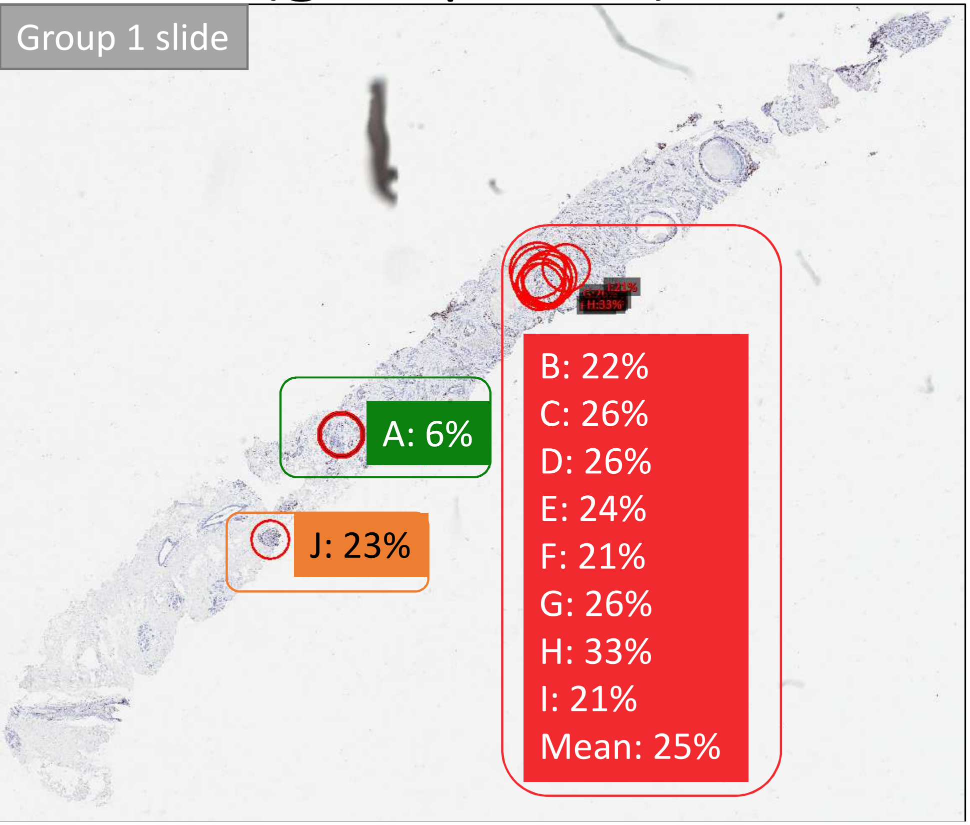

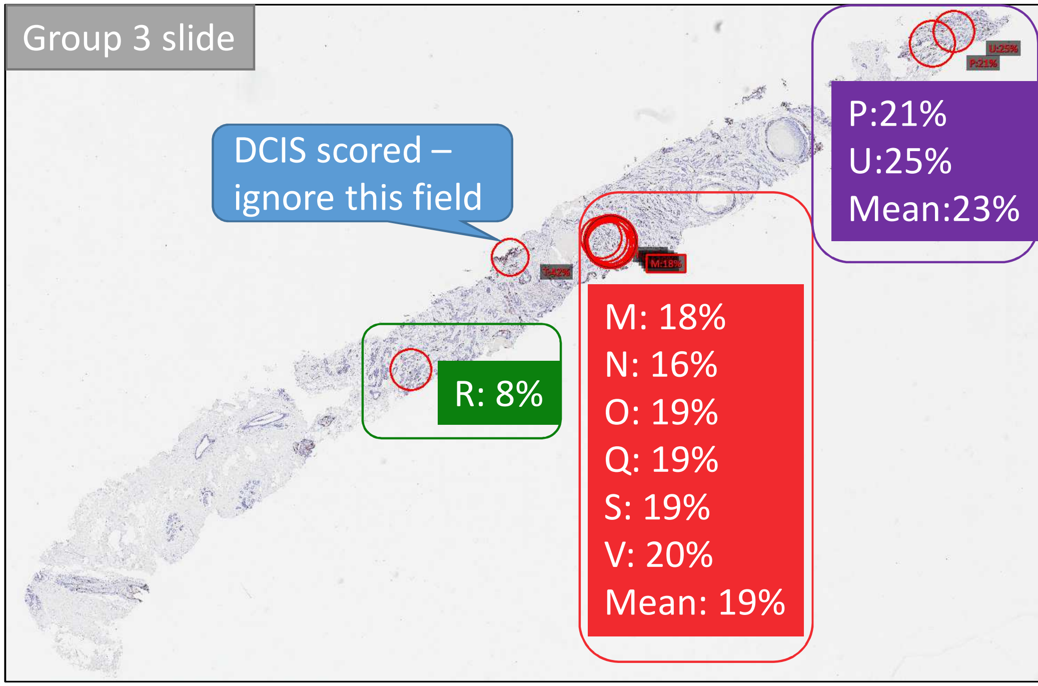

Both lab A (group 1) and R (group 3) scored considerably lower compared to other labs. Apparently, they've selected similar hot-spot fields (albeit in different sections).
